# Evaluation of quantitative biomarkers of aging in human PBMCs

**DOI:** 10.3389/fragi.2023.1260502

**Published:** 2023-09-15

**Authors:** Brady M. Owen, James Phie, Jennifer Huynh, Scott Needham, Cameron Fraser

**Affiliations:** Systematic Medicine, Melbourne, VIC, Australia

**Keywords:** aging, drug discovery, DNA methylation, apoptosis, proteostasis, cytokines

## Abstract

Functional decline with age contributes significantly to the burden of disease in developed countries. There is growing interest in the development of therapeutic interventions which slow or even reverse aging. Time and cost constraints prohibit the testing of a large number of interventions for health and lifespan extension in model organisms. Cell-based models of aging could enable high throughput testing of potential interventions. Despite extensive reports in the literature of cell properties that correlate with donor age, few are robustly observed across different laboratories. This casts doubt on the extent that aging signatures are captured in cultured cells. We tested molecular changes previously reported to correlate with donor age in peripheral blood mononuclear cells (PBMCs) and evaluated their suitability for inclusion in a panel of functional aging measures. The tested measures spanned several pathways implicated in aging including epigenetic changes, apoptosis, proteostasis, and intracellular communication. Surprisingly, only two markers correlated with donor age. DNA methylation age accurately predicted donor age confirming this is a robust aging biomarker. Additionally, the apoptotic marker CD95 correlated with donor age but only within subsets of PBMCs. To demonstrate cellular rejuvenation in response to a treatment will require integration of multiple read-outs of cell function. However, building a panel of measures to detect aging in cells is challenging and further research is needed to identify robust predictors of age in humans.

## 1 Introduction

Aging is the product of accumulated decline in function of an organism leading to disease and ultimately death. In high income countries, life expectancy has increased over the last 50 years but this has been accompanied by an increase in them number of years spent in poor health (Wang et al., 2020). The increased burden of disease is likely a combination of functional decline in cells, changes in the properties of the extracellular matrix, and tissue disorganisation. The relative contribution of each of these levels of organisation is not known and may vary between individuals. Models of aging are needed to improve our understanding of the aging process and test interventions which may slow the functional decline.

Cell-based systems have many advantages for the testing of geroprotective interventions including high reproducibility, high throughput, and low cost. However, robust measures of cellular aging are needed to evaluate the effectiveness of interventions. DNA methylation clocks have been used extensively to predict chronological age from cultured cells with high accuracy, but the clinical relevance is not clear. DNA methylation plays incompletely understood roles in gene expression, genomic stability and development (Greenberg and Bourc’his, 2019). Understanding of the relationship between DNA methylation age and health is limited and clock predictions can change transiently under stress conditions (Poganik et al., 2023). Therefore, whether DNA methylation clocks can capture the slowing or reversal of aging in cultured cells is unknown.

Measuring cell functions that are known to decline with age may complement DNA methylation clocks with readouts that are closer to the clinical manifestations of aging. Cell functions, spanning many pathways have been reported to change with age and these are summarised in Supplementary Table S3. However, some of these age correlations could not be reproduced in other populations or have never been independently validated (see Supplementary Table S3). A panel of cell functions that robustly correlate with donor age and span distinct molecular pathways could be a valuable readout for high throughput screening of interventions that slow or reverse aging.

In this study, we sought to validate a subset of previously reported aging measures on *ex vivo* peripheral blood mononuclear cells (PBMCs). The measures spanned distinct molecular pathways including epigenetic changes, apoptosis, proteostasis and cellular communication. Surprisingly, only DNA methylation clocks and CD95 expression in specific subsets of PBMCs correlated with donor age. Many features of aged cells have been reported in the literature. However, this study highlights these may not replicate for different populations and further research is needed to identify robust markers of aging in PBMCs.

## 2 Methods

### 2.1 Ethics approval

The study complied with the principles of the Declaration of Helsinki, and the use of human blood samples in this study was approved by the St Vincent’s Hospital Melbourne Human Research Ethics Committee (HREC 130/22). Informed consent was obtained from all human participants involved in this study prior to their participation. Confidentiality and privacy were ensured throughout the study, and all data collected were handled and stored in accordance with the ethics approval.

### 2.2 Study design

#### 2.2.1 Participant recruitment

For fresh blood samples, participants were recruited from a clinical trials database and by word of mouth. The following exclusion criteria were applied to potential participants in this study: individuals currently undergoing cancer or immune suppression treatment, those who have received a blood transfusion within the last 30 days or a vaccination within the last 7 days, individuals with genetic syndromes or blood borne diseases such as HIV, individuals who have been admitted to a hospital for a chronic condition within the last 3 months, and those currently experiencing an acute illness such as a cold or flu.

Remnant buffy coats from a random subset of blood donors in the Melbourne, Australia area were supplied by the Australian Red Cross Lifeblood service.

The demographic details of the blood donors in this study are provided in Table 1.

**TABLE 1:** Demographic information of blood donors.

	Fresh blood samples	Buffy coat remnant samples
Total	36	54
**Age**		
10–19	0	1
20–29	9	12
30–39	5	9
40–49	5	9
50–59	4	10
60–69	9	9
70–79	4	4
**Sex**		
Male	12	29
Female	24	25

#### 2.2.2 Blinding and randomisation

The processing order was randomised for all assays. All operators were blinded to donor age during the assays.

#### 2.2.3 Fresh blood samples

For these samples, PBMC extraction, flow cytometry, cytokine secretion assays and chymotrypsin-like proteasomal activity assays were performed on the day of blood collection. The aim was to minimise the time in culture and avoid freezing as this may change cell behaviour. It was impractical to complete the entire study in a single day, so blood collection and the corresponding assays were performed across four batches, each comprising roughly one-quarter of the study samples. For each donor, 36 mL of blood was collected in lithium heparin tubes by phlebotomists at Emeritus Research. A separate tube of blood was collected and frozen for DNA methylation arrays.

#### 2.2.4 Remnant buffy coat samples

Cytokine secretion and chymotrypsin-like proteasomal activity was validated on a second set of remnant buffy coats provided the day after blood collection. PBMCs were extracted immediately upon receipt then frozen. The cells were thawed and the assays for all samples were performed on a single day. This prevents experimental noise from batch effects, but the freezing and thawing process may introduce artifacts.

### 2.3 Peripheral blood mononuclear cells extraction

Blood samples were centrifuged at 1,000 *g* for 10 min in a 50 mL conical tube. The plasma was discarded before carefully pouring the buffy coat layer into a new 50 mL tube. The buffy coat was diluted 1:2 with PBS with 2% foetal bovine serum (FBS) (Thermo Fisher Scientific 26,140-079). All samples that were directly compared were extracted using the same FBS batch. For fresh samples, the entire buffy coat was used. For remnant samples, the volume exceeded what could be processed in one batch so 14 mL of buffy coat was used. PBMCs were isolated using SepMate-50 PBMC isolation tubes (Stem Cell Technologies 85,450) filled with 15 mL of Ficoll-Paque (Sigma Aldrich GE17-5442-02) according to the manufacturer’s instructions. The serum layer was aspirated and discarded without disrupting the PBMC layer. The PBMCs were rapidly poured into a new 50 mL tube. PBMCs were washed by resuspending the pellet in 40 mL of PBS with 2% FBS, centrifuging at 200 g for 8 min and discarding the supernatant. For remnant samples only, erythrocyte lysis was performed as described below. Both fresh and remnant PBMCs were then washed once more with 40 mL of PBS with 2% FBS.

Cells were resuspended in 2 mL (fresh samples) or 20 mL (remnant samples) of RPMI complete medium (RPMI 1640 with GlutaMAX and HEPES (Thermo Fisher Scientific 72,400,047), 10% FBS, 1 × MEM non-essential amino acids (Sigma M7145), 1 mM Sodium pyruvate (Thermo Fisher Scientific 11,360,070)). For the fresh samples, cells were counted after staining with trypan blue (Sigma Aldrich T8154) using the LUNA-II cell counter (Logos Biosystems). For the remnant samples, cells were stained with acridine orange and propidium iodide (Logos Biosystems F23001) and counted using the LUNA-FX7 cell counter (Logos Biosystems).

For remnant samples, cells were frozen in CryoStor CS10 (Stem Cell Technologies 07,930) according to the manufacturer’s instructions.

#### 2.3.1 Erythrocyte lysis

PBMCs extracted from some remnant samples had visible erythrocyte contamination. This had not been observed for the fresh samples. Differences in the cell populations may mask age correlated signals, for example, erythrocytes are expected to have lower proteasomal activity than PBMCs (Papanagnou et al., 2018). Therefore, the remnant samples were subjected to an erythrocyte lysis step. PBMC pellets were resuspended in 3 mL of 1x red blood cell lysis buffer (eBioscience 00-4300-54) and incubated for 5 min at room temperature. At the end of the incubation, 40 mL of PBS with 2% FBS was added to each tube to stop the lysis. Cells were pelleted by centrifuging at 200 g for 8 min and the supernatant was discarded. PBMCs were washed with 40 mL of PBS with 2% FBS.

#### 2.3.2 Platelet depletion

PBMCs purified by density gradient techniques may contain a 10–1000-fold excess of platelets (Casale and Kaliner, 1982). Washing the cells with low-speed centrifugation (200 g in this study) reduces but will not eliminate platelet contamination. Variable levels of platelets in the samples may prevent the identification of age-related signatures. Platelets have detectable proteasomal activity (El-Kadiry and Merhi, 2021) so platelet depletion was performed for the remnant PBMCs used for the proteasomal assay.

Platelets were depleted by magnetic activated cell sorting (MACS). One million PBMCs were pelleted by centrifugation at 300 *g* for 8 min. The pellet was resuspended in 100 μL of pre-cooled FACS buffer (1x MACS BSA (Miltenyi Biotec 130-091-376) in autoMACS rinsing solution (Miltenyi Biotec 130-091-222) with CD61 microbeads (Miltenyi Biotec 130-051-101). Platelets bound the beads for 15 min at 4°C. LS columns (Miltenyi Biotec 130-042-401) were washed with 2 mL of FACS buffer. The cell-bead mixture was diluted by addition for 400 μL of FACS buffer then the suspension was added to the column. The flow through was collected in a sterile 15 mL tube then passed through the column a second time. The column was washed with 2 mL of FACS buffer and the flow through collected in the same tube. Cells were pelleted by centrifugation at 300 *g* for 8 min, the supernatant discarded, then resuspended in 500 μL of complete RPMI.

### 2.4 Methylation arrays

For DNA methylation arrays, blood was collected into Tempus Blood RNA Tubes (Thermo Fisher Scientific 4342792). These tubes contain stabilization buffer which immediately lyses the cells. RNA was pelleted by centrifugation according to the manufacturer’s instructions. Genomic DNA remains in the supernatant. Previously, Ferrante et al. (2018) demonstrated the genomic DNA can be recovered from the supernatant using phenol:chloroform:isoamyl alcohol. Based on this, we modified the protocol for DNA extraction from whole blood using the QIAamp DNA Micro Kit (Qiagen 56304). This kit recommends using 100 μL of whole blood as input which is equivalent to 400 μL of Tempus RNA blood tube supernatant (the supernatant contains 1 part blood, 2 parts stabilization reagent and 1 part PBS). We added 40 μL of proteinase K and 400 μL of buffer AL to 400 μL of Tempus RNA blood tube supernatant. Samples were pulse vortexed for 15 s then incubated at 56°C for 10 min with shaking at 1500 RPM. After adding 200 µL of ethanol and pulse vortexing for 15 s, samples were incubated at room temperature for 3 min before loading 700 µL into a QIAamp MinElute column. Samples were centrifuge at 6,000 *g* for 1 min and the flow through discarded. The remaining sample was loaded on the same column and the column was centrifuged again at 6,000 g for 1 min. The wash and elution steps were performed according to the manufacturer’s instructions. Samples were eluted in a volume of 50 μL and typically had concentrations of 75–125 ng/μL (3,750–6,250 ng total recovery) as measured using the Qubit 1x broad range dsDNA kit (Thermo Fisher Scientific Q33266).

Illumina DNA MethylationEPIC arrays were performed by the Australian Genome Research Facility (AGRF). The sample positions on the arrays were randomised.

DNA methylation data was pre-processed and Noob normalised with Minfi version 1.46.0 (Aryee et al., 2014). Epigenetic age was determined from the resulting beta values using PC-Clocks (Higgins-Chen et al., 2022).

### 2.5 Flow cytometry

Immunophenotyping of PBMCs was performed using the following antibodies: CD3-AF700 (BD biosciences 557,943), CD8-BV605 (BD biosciences 564,115), CD56-PE (Miltenyi 130-113-312), CD19-APC-Vio770 (Miltenyi 130-113-643), CD14-FITC (Miltenyi 130-113-146), CD95-APC (Miltenyi 130-113-005). IgG matched controls were run in parallel: IgG2a kappa-APC (Miltenyi 130-113-269), REA IgG1 APC (Miltenyi 130-113-446), IgG1 k APC (Thermo scientific 17-4714-82).

FACS buffer was cooled on ice before use. The PBMCs were aliquoted at 100,000 cells per well of a 96-well U bottom plate then pelleted by centrifugation at 300 *g* for 8 min. The cells were resuspended in 50 µL of FACS buffer containing 1:5 diluted FcR blocking reagent (Miltenyi Biotec 130-059-901) then incubated for 10 min at 4°C. To each sample, 50 μL of antibody cocktail in FACS buffer was added and samples were incubated for 20 min at 4°C, protected from light. Cells were washed once with 100 μL of FACS buffer then resuspended in 90 μL of FACS buffer. Immediately before flow cytometry, 10 μL of 1 μg/mL DAPI (Sigma Aldrich D9542) was added to the sample. Data were acquired on a Beckman Coulter CytoFLEX flow cytometer using CytExpert (version 2.4) and analysed using FlowJo software (BD Biosciences).

A compensation matrix was applied to all samples prior to the flow acquisition to correct for fluorescent spill over. The matrix was generated using compensation beads (Thermo Scientific 01-3,333-42) individually stained with each of the 6 antibodies, and PBMCs stained with DAPI. Gating was manually performed for all antibodies except CD95-APC populations. A CD95-APC FMO control was used to set gates for all CD95 populations except for CD3^+^CD8^+^ cells, where two distinct CD95 populations were discernible. Data analysis was performed by an experienced investigator independent to the study and blinded to study objectives. An example of the gating strategy is provided in Supplementary Figure S1.

### 2.6 Proteasomal activity assays

20S chymotrypsin-like proteasomal activity was measured using the Promega Chromotrypsin-like Cell-Based Proteasome-Glo Assay (G8660), following the manufacturer’s instructions. For the fresh cells, 20,000 PBMCs in 100 µL of complete RPMI were seeded in a white 96 well plate and incubated at room temperature for 2 hours. The assay was miniaturised for the remnant samples and 5,000 PBMCs were seeded in 20 µL of complete RPMI in a black 384 well plate. Both cell dilutions were confirmed to fall within the linear range of the assay (data not shown). The Proteasome-Glo substrate was thawed and equilibrated at room temperature for at least 30 min before adding 20 µL to each well. The plates were briefly centrifuged and then mixed on a plate shaker at 700 RPM for 2 min. Samples were incubated at room temperature for 10 min and luminescence was measured using a Perkin Elmer Victor Nivo plate reader. To normalize the luminescence between plates, a PBMC standard was included on each plate. A proteasome inhibitor control (20 µL lactacystin) was included in initial experiments to confirm the detection of non-specific protease activity was negligible (data not shown).

### 2.7 Cytokine secretion

PBMCs at 3 × 10^6^ cells/mL were seeded with 500 μL per well in 24 well non-TC treated plates (Thermo Scientific 144530). Cells were stimulated by adding 2 μL of lipopolysacharise (LPS, InvivoGen tlrl-b5lps) and phytohemagglutinin (PHA, InvivoGen inh-phap) solution or endotoxin free water as a control. The final concentrations of LPS and PHA were 0.1 μg/mL and 10 μg/mL respectively. Cells were incubated at 37°C, 5% CO_2_ for 4 (for CCL3 and IL-6 measurement) or 18 (for IFNγ and IL-2) hours. At the end of the incubation, the supernatant was collected, centrifuged at 300 *g* for 10 min then transferred to a new tube to remove any cell contamination. Samples were snap frozen on dry ice and stored at −80°C until ELISAs were performed.

Secreted cytokines were quantified by ELISA according to the manufacturer’s instructions (Thermo Scientific, CCL3: 88-7035-86, IL-6: 88-7066-86, IFNγ: 88731686, IL-2: 88-7025-86).

### 2.8 Statistical analyses

Sample size calculations and data analysis was performed using R statistical software (version 4.1.3).

Sample size calculations were performed using pwr version 1.3–0. For Pearson’s correlation a minimum sample size of 29 was determined using the following parameters: r = 0.5, power = 0.8, alpha = 0.05. For t-tests of means a minimum sample size of 6 per group was determined using the following parameters: power = 0.8, alpha = 0.05, effect size estimated from past studies (Supplementary Table S1). To account for uncertainty in effect size estimates, the minimum sample size was increased by 50%. All comparisons between young and aged donors were performed with at least 6 samples per group.

Pearson’s correlation coefficients were calculated between donor age and tested variables. To compare the mean values between young and aged groups, two-sample t-tests were performed. The young and aged groups were defined as 20–30 and 65–75 years respectively. Benjamini–Hochberg multiple testing correction was applied to adjust *p*-values for multiple comparisons (Benjamini et al., 2009).

## 3 Results

### 3.1 DNA methylation clocks accurately predict donor age

**FIGURE 1:**
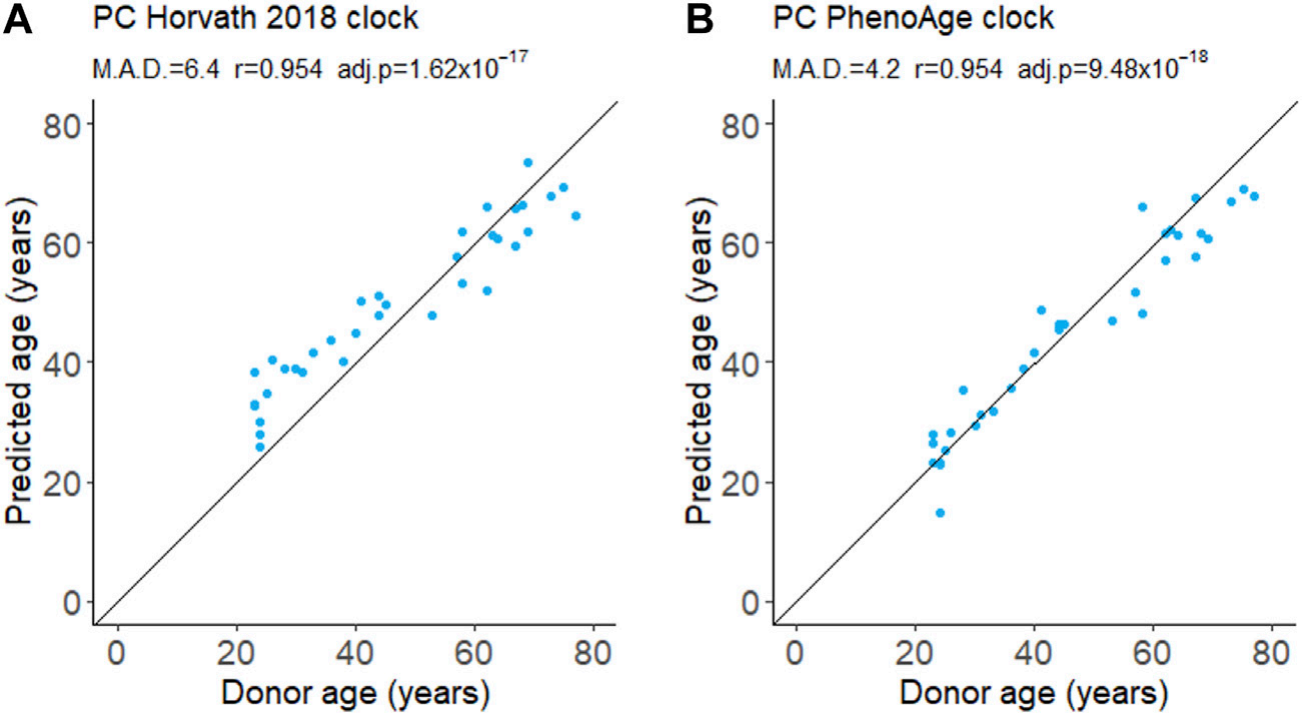
DNA methylation age from whole blood. Age was predicted using the first-generation PC Horvath 2018 clock **(A)** and the second-generation PC PhenoAge clock **(B)**. M.A.D. refers to mean absolute deviation in years. Pearson’s correlation coefficients (r) were calculated. adj.p refers to Benjamini–Hochberg adjusted *p*-values. N = 35.

We evaluated the performance of a first- and second-generation DNA methylation clock on fresh whole-blood samples. The Horvath 2018 is a first-generation chronological age predictor trained on skin and blood cells (Horvath et al., 2018). PhenoAge is a second-generation clock trained on phenotypic age estimated from blood-based biomarkers and is a predictor of mortality risk (Levine et al., 2018). We applied the updated principal component (PC) versions of these clocks which aggregate information from many CpGs to reduce the influence of technical noise at any one CpG (Higgins-Chen et al., 2022). Both the Horvath 2018 and PhenoAge clocks accurately predict the chronological age of donors (Figure 1). The Horvath 2018 clock predicted the chronological age with a mean absolute deviation (MAD) of 6.4 years while the PhenoAge clock had a MAD of 4.2 years. Consistent with previous reports, we also detected a modest decline in global DNA methylation levels with age (Supplementary Figure S2).

### 3.2 The proportion of CD3^+^CD8^+^ T cells expressing CD95 increases with age

Surface expression of the transmembrane glycoprotein CD95 is reportedly upregulated in some immune cells with age (Shinohara et al., 1995; Phelouzat et al., 1997; Aggarwal and Gupta, 1998; Jelaska and Korn, 1998; Potestio et al., 1998; Schindowski et al., 2000; Li et al., 2020). Interaction of CD95 with its ligand (CD95L) typically induces apoptosis although activation of other signalling pathways has also been reported (Seyrek et al., 2022). CD95 induced apoptosis plays a role in the elimination of self-reactive B and T lymphocytes during development and the elimination of activated immune cells after an infection has been resolved (Seyrek et al., 2022).

**FIGURE 2:**
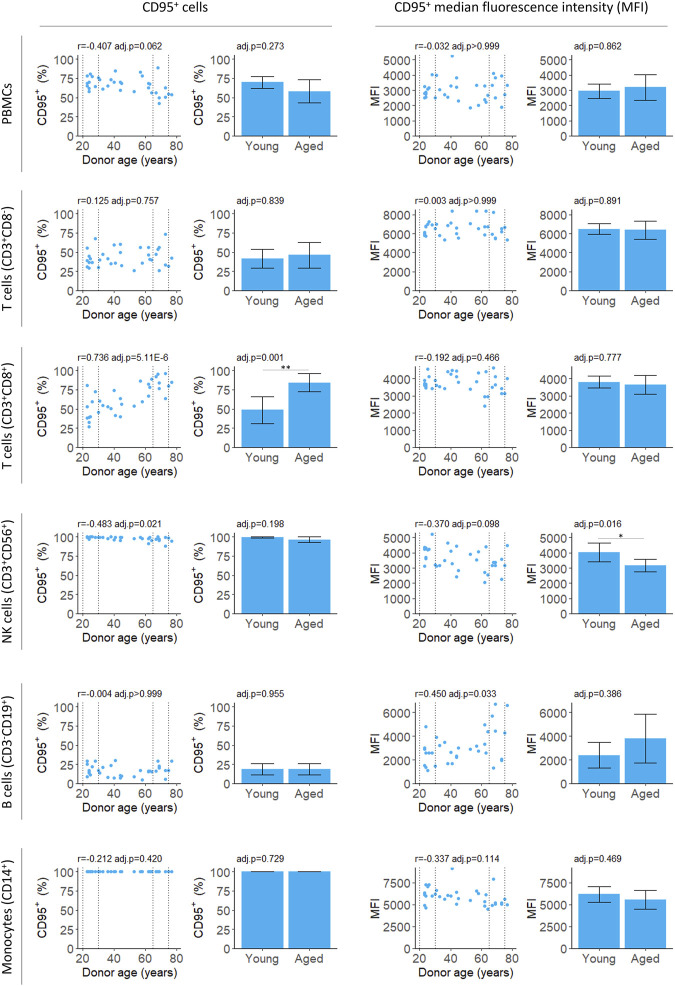
Flow cytometry analysis of CD95 expression by PBMCs isolated from fresh blood samples. CD95^+^ cells indicate the proportion of the cell population where CD95 was detected. CD95^+^ median fluorescence intensity (MFI) indicates the intensity of the CD95 signal on the CD95^+^ cells. For scatter plots, Pearson’s correlation (r) was calculated. Bar chart data is a subset of the data on the scatter plots (indicated by the dotted lines) and shows the mean and 95% confidence intervals for young (20–30 years) and aged (65–75 years) donors. *p*-values were determined using two-tailed t-tests. The number of samples for each analysis is provided in Table 2.

We measured both the proportion of PBMCs expressing CD95 and intensity of the CD95 signal on CD95^+^ cells (median fluorescence intensity, MFI) for PBMCs isolates from fresh blood samples. The proportion of CD95^+^ PBMCs decreased slightly with age although this was not significant after applying a multiple testing correction (Figure 2; Table 2). There was also no significant difference between the young and aged groups.

**TABLE 2:** Summary of relationship between biomarkers and aging. Data is collated from Figures 1–4. ^1^Performed for PBMCs isolated from fresh blood only. Results that were statistically significant, at a significance threshold of 0.05, are shown in bold.

Assay	Correlation			Young vs. aged	
	Pearson’s R [95% confidence interval]	Adjusted *p*-value	n	Adjusted *p*-value	n young, n aged
**Epigenetic age** ^1^					
PC Horvath 2018	0.954 [0.910, 0.977]	**1.62E-17**	35	**4.35E-08**	10, 7
PC PhenoAge	0.954 [0.910, 0.977]	**9.48E-18**	35	**1.06E-05**	10, 7
**Apoptosis** ^1^					
Population: PBMCs					
% CD95^+^	−0.407 [-0.655, −0.08]	0.062	34	0.273	10, 7
MFI	−0.032 [-0.366, 0.309]	>0.999	34	0.862	10, 7
Population: CD3^+^CD8^−^ T cells					
% CD95^+^	0.125 [-0.223, 0.444]	0.757	34	0.839	10, 7
MFI	0.003 [-0.335, 0.341]	>0.999	34	0.891	10, 7
Population CD3^+^CD8^+^ T cells					
% CD95^+^	0.736 [0.53, 0.86]	**5.11E-06**	34	**0.001**	10, 7
MFI	−0.192 [-0.498, 0.156]	0.466	34	0.777	10, 7
Population: CD3^+^CD56^+^ NK cells					
% CD95^+^	−0.483 [-0.706, −0.173]	**0.021**	34	0.198	10, 7
MFI	−0.370 [-0.629, −0.036]	0.098	34	**0.016**	10, 7
Population: CD3^−^CD19^+^ B cells					
% CD95^+^	−0.004 [-0.342, 0.334]	>0.999	34	0.955	10, 7
MFI	0.450 [0.132, 0.684]	**0.033**	34	0.386	10, 7
Population: CD14^+^ monocytes					
% CD95^+^	−0.212 [-0.513, 0.136]	0.420	34	0.729	10, 7
MFI	−0.337 [-0.606, 0.002]	0.114	34	0.469	10, 7
**20S proteasomal activity**					
Fresh	0.260 [-0.133, 0.583]	0.379	27	0.357	6, 7
Remnant	0.289 [0.015, 0.523]	0.097	51	**0.010**	12, 6
**Cytokine secretion**					
Fresh blood PBMCs					
CCL3	−0.119 [-0.465, 0.259]	0.790	29	0.892	7, 7
IL-6	−0.006 [-0.392, 0.382]	>0.999	26	0.800	6, 6
IFNγ	−0.412 [-0.685, −0.038]	0.090	27	0.492	6, 7
Remnant buffy coat PBMCs					
CCL3	0.014 [-0.271, 0.297]	>0.999	48	0.857	11, 7
IL-6	0.030 [-0.251, 0.306]	>0.999	50	0.793	12, 7

The proportion of CD3^+^CD8^+^ T cells expressing CD95 increased robustly with donor age, in agreement with previous studies (Shinohara et al., 1995; Phelouzat et al., 1997; Aggarwal and Gupta, 1998). Both the correlation and difference between young and aged groups were significant (Figure 2; Table 2). The proportion of B cells that were CD95^+^ was unchanged with age. However, there was an increase in MFI for the CD95^+^ cells suggesting the level of receptor expression increases with age (Figure 2; Table 2).

In young donors, near 100% of natural killer (NK) T cells expressed CD95. There was a significant decline in CD95^+^ NK T cells with age although the effect size was very small and the difference between young and aged groups was not significant (Figure 2; Table 2). The CD95 MFI trended down with age for NK T cells and the signal was significantly lower for aged donors compared to young donors. This suggests CD95 expression may decline with age in NK T cells, although this requires further validation, and the effect size is likely to be very small.

We found no relationship between the proportion of any cell type and age (Supplementary Figure S2; Supplementary Table S2).

The age dependent CD95 expression by CD3^+^CD8^+^ T cells is an interesting area for future research. There is a potential for more extensive apoptosis after T cell activation. However, activation CD95 receptors only leads to apoptosis in some contexts (Zhang et al., 1996; Varadhachary et al., 1997). Direct measurements of apoptosis and cell survival after T cell activation are needed to determine if this contributes to the decline in immune function reported with age.

### 3.3 Chymotrypsin-like proteasomal activity does not correlate with donor age

The proteasome is a crucial cellular machinery which degrades damaged proteins, eliminates transient proteins, and produces peptides for antigen presentation on MHC class II molecules (Kumar Deshmukh et al., 2019). Proteasomal activity is reported to decline with age in several cell types (Hwang et al., 2007; Papanagnou et al., 2018; Sarkar et al., 2020). This may contribute to aging related pathologies such as cancer, cardiovascular disease, and neurodegenerative diseases which are associated with proteasomal impairment (Chondrogianni et al., 2014; Opoku-Nsiah and Gestwicki, 2018).

**FIGURE 3:**
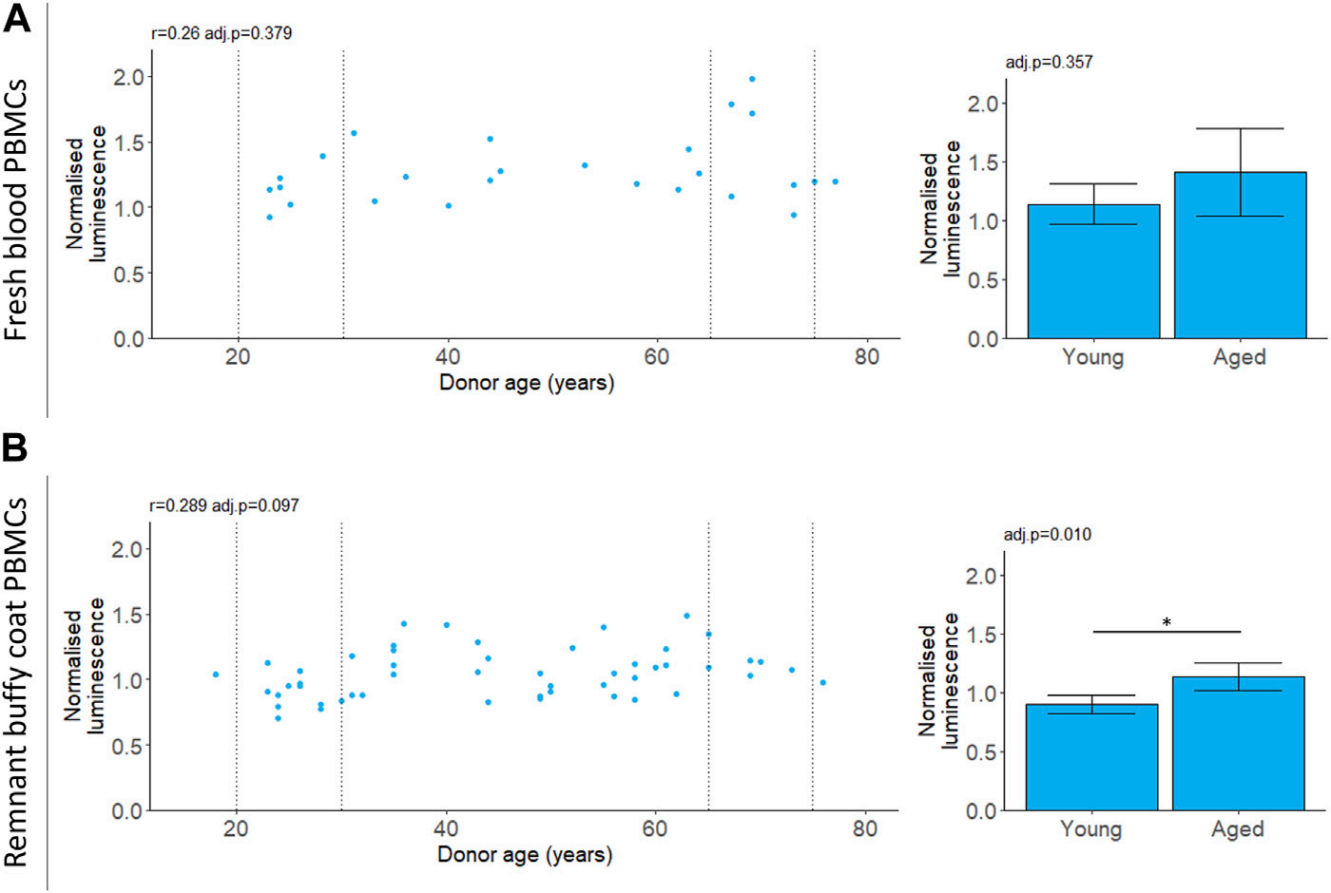
20 S chymotrypsin-like proteasomal activity in aging. Chymotrypsin-like proteasomal activity was measured in PBMCs extracted from fresh blood **(A)** or remnant buffy coats **(B)**. Luminescence values are normalised to a control PBMC sample which was not included in the analysis. Scatter plots represent the mean of three technical replicates for each sample and Pearson’s correlations (r) were calculated for the data. Bar chart data is a subset of the data on the scatter plots (indicated by the dotted lines) and shows the mean and 95% confidence intervals for young (20–30 years) and aged (65–75 years) donors. *p*-values were determined using two-tailed t-tests. The number of samples for each analysis is provided in Table 2.

We measured chymotrypsin-like proteasomal activity in PBMCs which will capture the cumulative activity of both the proteasome and the immunoproteasome (Johnston-Carey et al., 2016; Murata et al., 2018; Kumar Deshmukh et al., 2019). For PBMCs isolated from fresh blood, chymotrypsin-like proteasomal activity did not depend on donor age (Table 2; Figure 3). For the buffy coat remnant samples, mean proteasomal activity was higher for donors aged 65–75 compared to donors aged 20–30 (Table 2; Figure 3). This is in contrast to the age-dependent decline in proteasomal activity previously reported for PBMCs (Papanagnou et al., 2018) and is not reflected in the correlation (Table 2; Figure 3).

### 3.4 Cytokine secretion does not correlate with donor age

Cytokines are signalling proteins that play a crucial role in inflammation and the immune response. Cytokines are dysregulated with age and this may contribute to chronic inflammatory diseases (Rea et al., 2018).

We stimulated PBMCs with LPS and PHA and measured secreted pro-inflammatory cytokines IL-6, CCL3, IL-2 and IFNγ. IL-6, IL-2 and IFNγ were selected based on previous reports that secretion of these cytokines correlated with donor age (Supplementary Table S3). CCL3 was included as it is secreted by many types of PBMCs and has been implicated in cancer and other diseases (Bhavsar et al., 2015).

**FIGURE 4:**
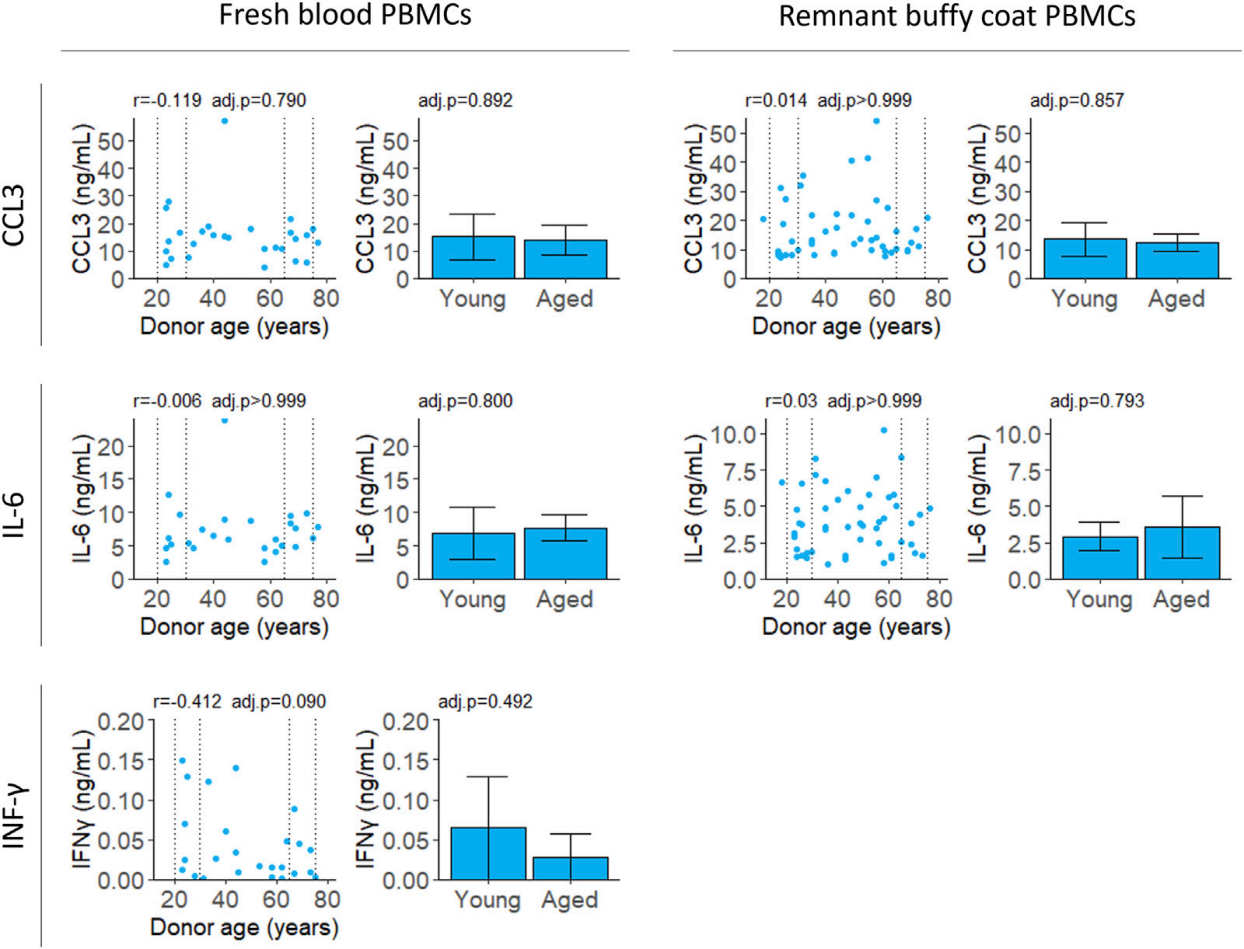
Cytokine secretion after stimulation with LPS and PHA. Cell supernatant was collected 4 (CCL3 and IL-6) or 18 (IFNγ) hours after stimulation and cytokine levels were measured by ELISA. Scatter plots represent the mean of three technical replicates for each sample and Pearson’s correlations were calculated. Bar plots are constructed from a subset of the scatter plot data (indicated by the dotted lines) and represent the mean and 95% confidence interval for young (20–30 years) and aged (65–75 years) groups. *p*-values were determined using two-tailed t-tests. The number of samples for each analysis is provided in Table 2.

All cytokines were measured for PBMCs isolated from fresh samples and IL-6 and CCL3 were validated in PBMCs from remnant buffy coats. Secreted levels of IL-2 were below the limit of detection at 18 h (data not shown). Unstimulated cells secreted extremely low levels of CCL3, IL-6 and IFNγ (Supplementary Figure S4). There was no correlation with age for any of the cytokines tested (Figure 4; Table 2).

## 4 Discussion

Using cell culture for drug discovery requires measures of the aged phenotype that are robust and amenable to high throughput screening. In this study, we tested four distinct measures of aging that were reported in the literature. We were only able to replicate a correlation with age for two of them: DNA methylation and the apoptosis marker CD95; albeit only in a subset of PBMCs (Table 2).

### 4.1 Challenges identifying cellular predictors of age

There are obvious visual and functional differences between young and aged individuals at the whole organism level. However, recapitulation of these differences in cell culture has proven difficult. Potential reasons for this are discussed below.

#### 4.1.1 Is cell dysfunction the main driver of aging?

Aging is a complex process influenced by both intrinsic cellular factors and the surrounding tissue environment. It may be difficult to identify cellular markers of aging if these are heavily influenced by extrinsic factors like cell signalling, nutrient availability, and waste accumulation (López-Otín et al., 2023). Heterochronic parabiosis and plasma exchange experiments demonstrate cells can be rejuvenated by exposure to young plasma or by dilution of old plasma with saline (Villeda et al., 2011; Conboy and Rando, 2012; Elabd et al., 2014; Rebo et al., 2016; Kang and Yang, 2020; Mehdipour et al., 2020; Kim et al., 2022). A cell culture model of aging must acknowledge that both the breakdown in cellular functions and the tissue environment are important.

The effect of body environment may explain why IL-6 secretion by PBMCs correlates with age in some studies but not others (Supplementary Table S3). In agreement with this work (Figure 4), Beharka et al. (2001) reported no relationship between age and IL-6 secretion after stimulation with PHA. They hypothesised inflammatory conditions were more common in older subjects meaning the previously reported age correlations may have occurred due to lack of strict exclusion criteria during participant selection.

These findings emphasize the importance of considering the tissue and organismal context when studying age-related changes in cells, as the tissue environment and extrinsic factors can heavily influence the aging process.

#### 4.1.2 Is any single driver of aging powerful enough to capture?

Aging is a stochastic and heterogeneous process, making it difficult to capture with a single biomarker. There are many different causes of death in humans and even genetically similar model organisms have differences in health and lifespan (Vanhooren and Libert, 2013; Zhao et al., 2017). While many cellular systems decline with age, the rate of decline in each system likely varies by individual (Rando and Wyss-Coray, 2021).

Effective biomarkers of aging may need to integrate signals from multiple aging pathways. DNA methylation clocks use methylation levels at hundreds of CpGs which may capture signals from different aging processes (Liu et al., 2020; Levine et al., 2022). Consistent with this, two different DNA methylation clocks robustly predicted donor age from the samples in this study (Figure 1). The correlation of 0.95 (Figure 1) greatly exceeded the next best age predictor, the proportion of CD3^+^CD8^+^ T cells that expressed CD95, with a correlation of 0.74 (Figure 2).

Recently, transcriptomic clocks have been developed which also integrate information from many cell functions and may be closer to the clinical endpoint (Remondini et al., 2017; Fleischer et al., 2018; Janssens et al., 2019). However, these have three limitations. Transcriptomic clocks are less accurate than DNA methylation clocks (Jylhävä et al., 2017), mRNA levels are difficult to measure reliably between laboratories (Sprang et al., 2022) and mRNA levels are an imperfect predictor of protein levels (Takemon et al., 2021).

Integration of multiple functional measures which correlate imperfectly with age may result in an accurate age predictor that also provides insights into clinical outcomes. The proportion of CD3^+^CD8^+^ T cells that express CD95 could be one component of this panel of measures but identifying additional robust biomarkers that correlate with age requires additional research.

#### 4.1.3 How old is a cell from an aged donor?

Cell age and donor age are often not interchangeable. Cells such as neurons and cardiomyocytes lose their replicative potential early in development so the cells are approximately the same age as the organism (Anda et al., 2016; Foglia and Poss, 2016). However, tissues with regenerative capacity contain a mix of cells that may be as old as neurons and those which have been replaced (Drigo et al., 2019). Cell division can dilute damaged cellular components, but it is unclear if this improves cell function. This likely depends on both the cell type and the functional property that is being measured.

Cell turnover could be particularly relevant for measurements of proteasomal activity. Most studies that report a decline in proteasomal activity with age were performed in dermal fibroblasts. Fibroblasts divide infrequently in undamaged skin (Plikus et al., 2021), so the proteasome and autophagy are likely important for maintenance of protein quality. In contrast, the PBMCs used in this study are a mixed population of cells with dramatically different circulation lifespans, ranging from a few days for monocytes (van Furth and Cohn, 1968) and naive B cells (Macallan et al., 2005) to multiple years for B memory cells (Crotty et al., 2003; Jones et al., 2015) and some T cells (Tough and Sprent, 1995). Proteasomal activity likely varies between cell types and may be affected by how recently the cell last divided.

When sampling tissues with proliferative potential, it is important to consider that the cells will vary in age. Variations in cell division history due to genetic and environmental factors unique to each donor add another layer of complexity which may impact measures of aging.

#### 4.1.4 Does cell culture mask aging signatures?

Cells are cultured in specific culture media, and under set temperature and carbon dioxide levels. Typically, these mimic physiological conditions but subtle differences exist between laboratories which may explain the lack of consensus for many measurements in the literature (Supplementary Table S3). The effect of cell culture is tightly coupled to the extent that the features of aging are intrinsic to the cells. The more dependent they are on the cellular environment, the more modifiable they will be by culture conditions. Three ways culture conditions may mask aging signatures are discussed below.

Age specific differences in cell populations may be lost due to selection. The PBMCs used in these experiments will be enriched for characteristics that facilitate survival in culture. Certain cell types, such as neutrophils, have limited survival capacity in culture (Blanter et al., 2021). Within each cell type, there are likely to be subpopulations of cells that are better adapted to culture conditions. The loss of certain subpopulations or change in the cell ratios could lead to loss of aging signatures. Selection effects will become more pronounced with prolonged culture. One reason PBMCs were selected for this study is they can be used *ex vivo* without the need for propagation as 1 × 10^6^ PBMCs per millilitre of whole blood can be recovered from a typical donation (Corkum et al., 2015). For the fresh samples, all experiments were performed on the day of blood collection to further minimise culture artifacts.

Culture conditions may cause loss of age-related differences between cells. For instance, cell culture may replicate the conditions of young plasma exchange which causes rejuvenation of aged organisms (Rebo et al., 2016). Cells are removed from aged blood, which may contain drivers of cellular dysfunction, and placed in culture media. Rejuvenating components of young blood may be found in foetal bovine serum which is often used as a supplement in culture media. Subtle differences in culture media may explain why aging changes often do not reproduce between laboratories (Table 2; Supplementary Table S3).

Cell culture conditions may not provide the appropriate stimulus for certain measures to exhibit age-related differences. For instance, proteasomal activity is upregulated in the absence of essential amino acids (Vabulas and Hartl, 2005). In this study, measurements of chymotrypsin-like proteasomal activity were made on live PBMCs in cell culture media with an abundance of amino acids. All donors had similar levels of proteasomal activity regardless of age (Figure 3). These results are in contrast with previous studies that showed a decline in proteasomal activity with age in PBMCs (Papanagnou et al., 2018). Papanagnou et al. (2018) measured proteasomal activity in lysed cells without amino acid supplementation. The reported decline in proteasomal activity with age (Hwang et al., 2007; Papanagnou et al., 2018; Sarkar et al., 2020) may only be apparent when the system is challenged by low levels of amino acids.

#### 4.1.5 Which cells in the body are aging?

The manifestations of aging may vary between cell types. The goal of this study was to identify general mechanisms of aging that are expected to occur in many cell types. The rationale behind this was it would allow for screening of interventions that have the potential to restore function to multiple cell types.

We chose PBMCs as a heterogeneous cell population to enrich for aging signatures that occur in more than one cell type. PBMCs interact with most cells in the body. They secrete cytokines that regulate inflammation and wound healing (Zhang and An, 2007) and respond to signals from cancerous or senescent cells in tissues (Langhi Prata et al., 2018; Xia et al., 2021). Therefore, we hypothesised that PBMCs may be representative of, or even a causal driver of age-related decline in many tissues.

A limitation of using a heterogenous cell population is this may cause increased noise in the measures of aging. Using a highly purified cell population and studying a cell-type specific function may give stronger aging signals. Indeed, we found the proportion of CD95^+^ cells correlated with age for CD3^+^CD8^+^ T cells but not for a mixed population of PBMCs (Figure 2; Table 2). When working with any single cell type, there is a risk of identifying features of aging that are cell-type specific. For aging drug discovery, validation of interventions in multiple cell types is essential to confirm a general effect on aging.

### 4.2 Implications for drug discovery

Cell culture systems offer the advantage of low cost and high throughput screening for potential aging interventions. However, to conduct meaningful screens, it is crucial to have a model in which age reversal can be measured. The accurate chronological age predictions of epigenetic clocks present an attractive tool for such screens. However, confidence in the use of epigenetic clocks is still lacking due to the unclear relationship between methylation age and clinical outcomes.

Here we have shown that some reported age-related changes in cells may not reproduce on different populations. Our goal was to perform all assays on the same blood samples so we could only test a small subset of the measures reported in the literature. Further investigation is needed to determine if other measures may be more reliable.

At present, there is a lack of reliable measures of aging in cell culture. Due to the complex, multifaceted nature of aging, it is plausible that few robust signatures of aging are present in cells in culture. Instead aging may be better studied using whole organism models such as *Drosophila melanogaster*, *Caenorhabditis elegans* or *Mus musculus*. The potential to perform high throughput screens for aging interventions is an exciting area of research. However, the success of these programs is critically dependent on high-quality models of aging.

## Data Availability

DNA methylation data is deposited in the Gene Expression Omnibus (GEO) repository with the accession number GSE235717.
