# Supplementary material for: Evaluation of quantitative biomarkers of aging in human PBMCs
Source: Front Aging. 2023 Sep 15;4:1260502. doi: 10.3389/fragi.2023.1260502 (PMC10540680; doi:10.3389/fragi.2023.1260502)
Supplement: Supplementary file 1 [file DataSheet1.pdf]

Supplementary Material

1 Supplementary figures

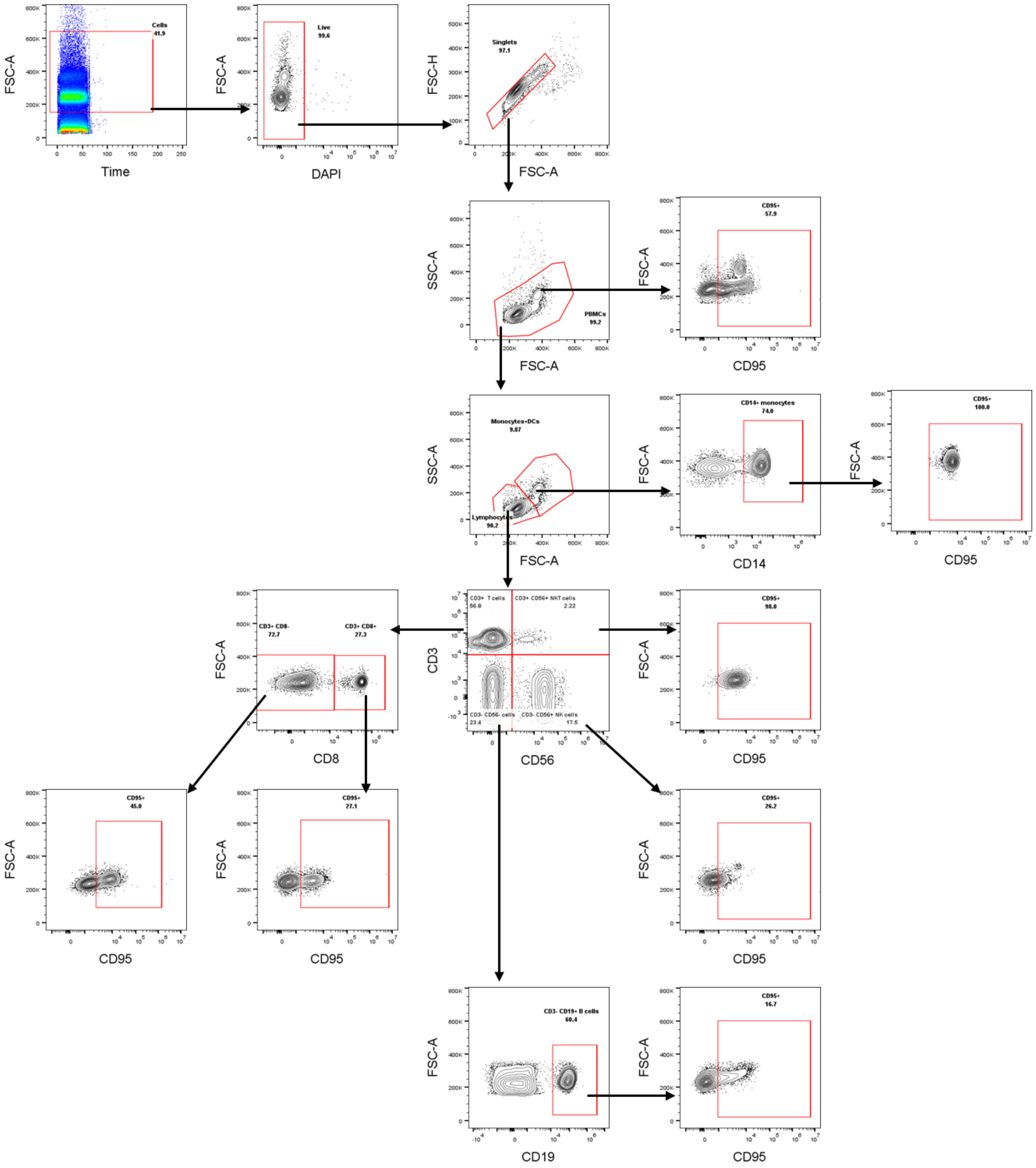

Supplementary Figure 1: Flow cytometry gating strategy.

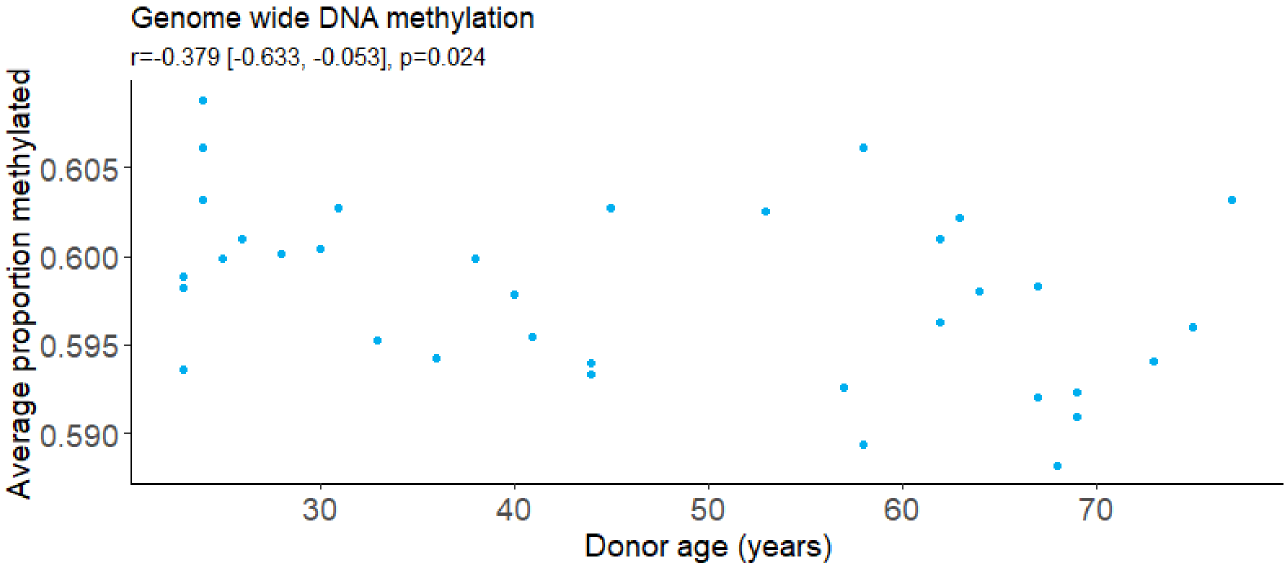

Supplementary Figure 2: Change in bulk DNA methylation levels with age. Pearson's R and 95 % confidence interval is given.

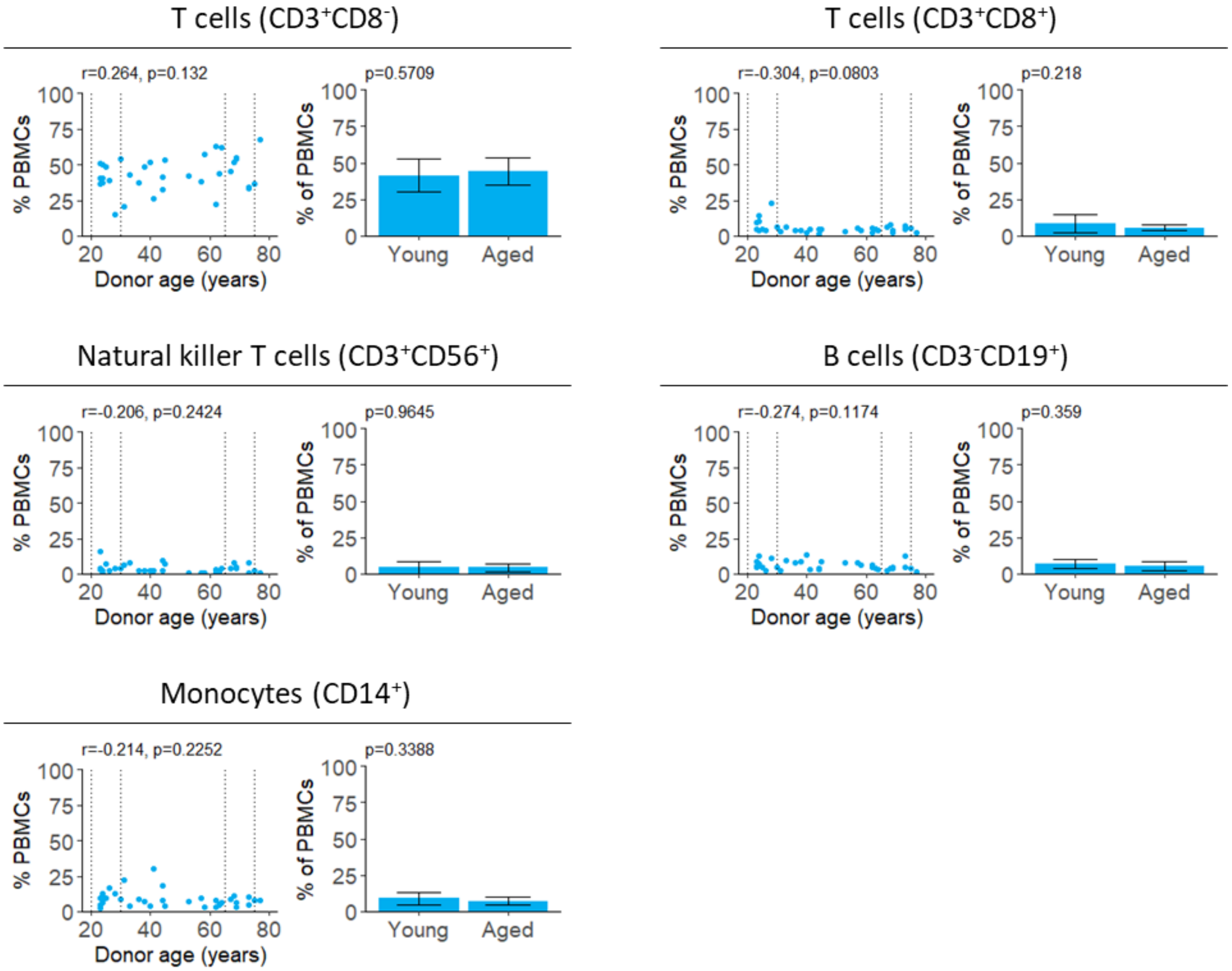

Supplementary Figure 3: Changes in cell populations with age. For scatter plots, Pearson's correlation (r) was calculated. The bar chart data is a subset of the data on the scatter plots (indicated by the dotted lines) and shows the mean and 95 % confidence intervals for young (20 – 30 years) and aged (65 – 75 years) donors, n=10 per group for young and n=7 per group for aged. Young and aged groups were compared using two-tailed t-tests.

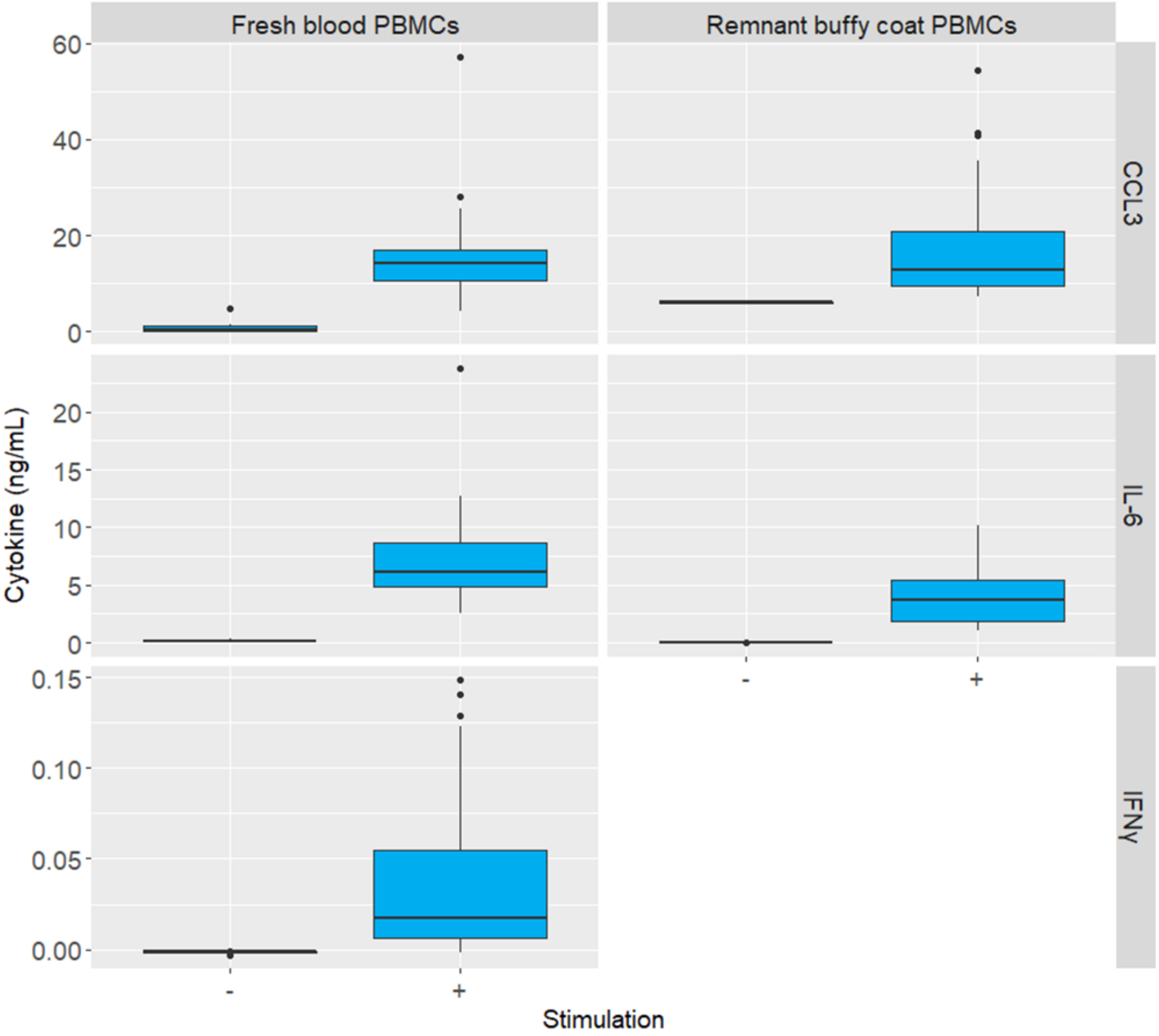

Supplementary Figure 4: Cytokine secretion in the presence (+) and absence (-) of LPS (0.1 µg/mL) and PHA (10 µg/m) stimulation.

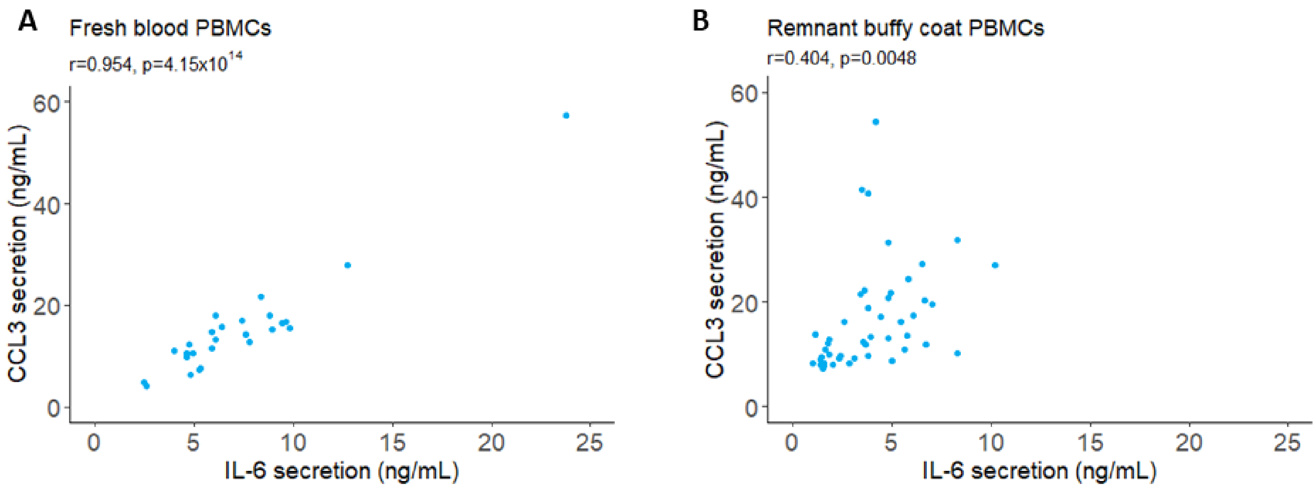

Supplementary Figure 5: Correlation between IL-6 and CCL3 secretion. Data from Figure 4 was plotted to show the relationship between IL-6 and CCL3 for each donor. Points represent the mean of three technical replicates. Pearson's correlation (r) was calculated.

2 Supplementary tables

Supplementary Table 1: Sample size calculations for comparison between young and aged donors using two-tailed t-test of means. The following parameters were used: power=0.8, alpha=0.05.

Experiment	Young group (years)	Aged group (years)	Effect size (Cohen's d)	Sample size (per group)	Reference
IL-2	16-19	45-55	3.0	4	(Li et al., 2020)
IFNγ	16-19	45-55	3.5	3	(Li et al., 2020)
IL-6	21-26	90-92	3.9	3	(Wolf et al., 2012)
CD95	<35	>60	4.0	3	(Schindowski et al., 2000)
Proteasomal activity	25-35	60-70	3.6	3	(Sarkar et al., 2020)

Supplementary Table 2: Pearson's correlations for changes in cell populations with age.

**Cell markers**	**Description**	**Pearson's R [95 % confidence interval]**	**p-value**
$CD14^+$	Monocytes	-0.214 [-0.515, 0.134]	0.225
$CD3^+CD56^+$	Natural killer T cells	-0.206 [-0.509, 0.142]	0.242
$CD3^+CD8^+$	T cells	-0.304 [-0.582, 0.038]	0.080
$CD3^+CD8^-$	T cells	0.264 [-0.082, 0.552]	0.132
$CD3^-CD19^+$	B cells	-0.274 [-0.560, 0.071]	0.117

Supplementary Table 3: Considered aging measures. Age prediction by epigenetic clocks has been independently validated in many studies and is not included in this table.

Assay	Cell type	Groups	Change with age	Reference
Epigenetic alterations				
CBX3 (HP1γ) immunocytochemistry	Dermal fibroblasts	60-70, n=8<br>25-35, n=3	Decrease	(Sarkar et al., 2020)
CBX3 (HP1γ) immunocytochemistry	Endothelial cells	45-60, n=7<br>25-35, n=3	Decrease	(Sarkar et al., 2020)
CBX3 (HP1γ) immunocytochemistry	Dermal fibroblasts	71-83, n=3<br>11, n=3	Decrease	(Miller et al., 2013)
H2A.J levels by fluorescence microscopy	Keratinocytes	18-90 years, n=53	Increased	(Rübe et al., 2021)
H3K9me3 immunocytochemistry	Dermal fibroblasts	60-70, n=8<br>25-35, n=3	Decrease	(Sarkar et al., 2020)
H3K9me3 immunocytochemistry	Endothelial cells	45-60, n=7<br>25-35, n=3	Decrease	(Sarkar et al., 2020)
H3K9me3 immunocytochemistry	Dermal fibroblasts	71-83, n=3<br>11, n=3	Decrease	(Miller et al., 2013)
Genome instability				
DNA double stranded breaks by γH2AX immunocytochemistry	Dermal fibroblasts	71-83, n=3<br>11, n=3	Increase	(Miller et al., 2013)
DNA double stranded breaks by γH2AX immunocytochemistry after bleomycin treatment	Dermal fibroblast	2- 92 years, n=9	Increase<br>R = 0.50	(Phillip et al., 2017)
DNA double stranded breaks by γH2AX immunocytochemistry after bleomycin treatment (signal localisation)	Dermal fibroblast	2- 92 years, n=9	Increase<br>R = 0.20	(Phillip et al., 2017)
LAP2α immunocytochemistry	Dermal fibroblasts	25-35, n=3<br>60-70, n=8	Decrease	(Sarkar et al., 2020)
LAP2α immunocytochemistry	Dermal fibroblasts	11, n=3<br>71-83, n=3	Decrease	(Miller et al., 2013)

LAP2α immunocytochemistry	Endothelial cells	25-35, n=3<br>45-60, n=7	Decrease	(Sarkar et al., 2020)
Nuclear entropy	Dermal fibroblast	2- 92 years, n=9	Increase<br>R = 0.37	(Phillip et al., 2017)
Nuclear size	Dermal fibroblast	2-92, n=9	Increased<br>R = 0.89	(Phillip et al., 2017)
Nuclear skewness	Dermal fibroblast	2- 92 years, n=9	Increase<br>R = 0.58	(Phillip et al., 2017)
Telomere length				
Expression of hTERT mRNA upon LPS stimulation	PBMCs	20-64, n=30	Decreased<br>R=-0.446, p=0.007	(Slusher et al., 2019)
Expression of hTERT mRNA upon PTX3 and LPS stimulation	PBMCs	57 ± 7, n=15<br>25 ± 3, n=15	Decreased<br>p = 0.033	(Slusher et al., 2019)
Telomere associated BP53	Keratinocytes	18-90 years, n=53	Increased	(Rübe et al., 2021)
Telomere length by FISH	Dermal fibroblasts	60-70, n=8<br>25-35, n=3	No change	(Sarkar et al., 2020)
Telomere length by FISH	Endothelial cells	45-60, n=7<br>25-35, n=3	No change	(Sarkar et al., 2020)
Telomere length by IQ-FISH	Keratinocytes	18-90 years, n=51	No change	(Rübe et al., 2021)
Telomere length by IQ-FISH	Lymphocytes	18-90 years, n=51	No change	(Rübe et al., 2021)
Telomere length by PCR	PBMCs	57 ± 7, n=15<br>25 ± 3, n=15	Decreased<br>p = 0.011	(Slusher et al., 2019)
Telomere length by PCR	PBMCs	20-64, n=30	Decreased<br>R = -0.404, p = 0.027	(Slusher et al., 2019)
Telomere length by quantitative FISH	Peripheral blood lymphocytes	60-69<br>70-79<br>80-89	Decreased<br>Male:<br>R = -0.495	(Canela et al., 2007)

		90-99<br>n=198 total	$p < 0.001$<br>Female:<br>$R = -0.335$<br>$p < 0.001$	
Apoptosis				
Apoptosis after induction with 2-deoxy-d-ribose	Lymphocytes	26.4 ± 5.1, n=34<br>73.8 ± 8.3, n=32	Increased<br>3.0-7.92%<br>$p < 0.05$	(Schindowski et al., 2000)
Apoptosis after induction with staurosporine	Lymphocytes	26.4 ± 5.1, n=34<br>73.8 ± 8.3, n=32	No change	(Schindowski et al., 2000)
Apoptosis detected by flow cytometry with 7-aminoactinomycin D after PHA and IL-2 stimulation	CD3+ CD45RO-	31 ± 3, n=7<br>67 ± 8, n=7	Increased<br>$p < 0.01$	(Herndon et al., 1997)
Apoptosis detected by flow cytometry with 7-aminoactinomycin D after PHA and IL-2 stimulation	CD3+ CD45RO+	31 ± 3, n=7<br>67 ± 8, n=7	No change	(Herndon et al., 1997)
Apoptosis detected by flow cytometry with 7-aminoactinomycin D after PHA and IL-2 stimulation	CD3+ T cells	31 ± 3, n=7<br>67 ± 8, n=7	No change	(Herndon et al., 1997)
Apoptosis detected by fluorescein-digoxigenin end-labelling of fragmented DNA.	Dermal fibroblasts	Foreskin, n=2<br>Adult, n=2	Increased	(Jelaska and Korn, 1998)
Apoptosis induction after anti-Fas antibody treatment – Propidium iodide staining	CD4+ T cells	20-29, n=10<br>65-95, n=10	Increased<br>26-55 %<br>$p < 0.001$	(Aggarwal and Gupta, 1998)
Apoptosis induction after anti-Fas antibody treatment – Propidium iodide staining	CD8+ T cells	20-29, n=10<br>65-95, n=10	Increased<br>18-50 %<br>$p < 0.001$	(Aggarwal and Gupta, 1998)
Apoptosis induction after anti-Fas antibody treatment – TUNEL assay	CD4+ T cells	20-29, n=10<br>65-95, n=10	Increased<br>24-64 %<br>$p < 0.001$	(Aggarwal and Gupta, 1998)
Apoptosis induction after anti-Fas antibody treatment – TUNEL assay	CD8+ T cells	20-29, n=10<br>65-95, n=10	Increased<br>22-56 %<br>$p < 0.001$	(Aggarwal and Gupta, 1998)
Apoptotic bodies after anti-Fas antibody treatment measured with Hoechst dye	Lymphocytes	20-29<br>65-95	Increased	(Aggarwal and Gupta, 1998)

Apoptotic cells detected by propidium iodide stain and flow cytometry.	Lymphocytes	21-60, n=40<br>66–80, n=24	Increased<br>7.2-9.3 %	(Potestio et al., 1998)
Apoptotic cells detected by propidium iodide stain and flow cytometry after PHA treatment.	Lymphocytes	21-60, n=40<br>66–80, n=24	Increased	(Potestio et al., 1998)
Apoptotic cells detected by propidium iodide stain and flow cytometry after α-CD3 treatment.	Lymphocytes	21-60, n=40<br>66–80, n=24	Increased	(Potestio et al., 1998)
Apoptotic cells detected by propidium iodide stain and flow cytometry.	Lymphocytes	26.4 ± 5.1, n=34<br>73.8 ± 8.3, n=32	Increased<br>0.84-1.35 %<br>$p < 0.05$	(Schindowski et al., 2000)
Apoptotic cells detected by propidium iodide stain and flow cytometry after α-CD95 treatment.	Lymphocytes	26.4 ± 5.1, n=34<br>73.8 ± 8.3, n=32	Increased<br>$p < 0.001$	(Schindowski et al., 2000)
Bax mRNA by qPCR	Lymphocytes	20-29, n=2<br>65-95, n=2	Increased<br>0.37-0.62 x β-actin	(Aggarwal and Gupta, 1998)
Bcl-2 levels by ELISA	PBMCs	26.4 ± 5.1, n=34<br>73.8 ± 8.3, n=32	No change	(Schindowski et al., 2000)
Bcl-2 levels by ELISA after activation with PHA and IL-2	PBMCs	26.4 ± 5.1, n=34<br>73.8 ± 8.3, n=32	Decreased<br>$p < 0.05$	(Schindowski et al., 2000)
Bcl-2 mRNA by qPCR	Lymphocytes	20-29, n=2<br>65-95, n=2	Decreased<br>0.29-0.14 x β-actin	(Aggarwal and Gupta, 1998)
Bcl-X_L mRNA by qPCR	Lymphocytes	20-29, n=2<br>65-95, n=2	No change	(Aggarwal and Gupta, 1998)
CD95 mRNA by qPCR	PBMCs	20-29<br>65-95	Increased<br>0.45-1.06 x β-actin	(Aggarwal and Gupta, 1998)
DNA fragmentation after anti-Fas antibody treatment	Lymphocytes	20-29, n=10<br>65-95, n=10	Increased	(Aggarwal and Gupta, 1998)
Flow cytometry measurement of Bcl-2	CD4+ T cells	20-29, n=10<br>65-95, n=10	Decreased<br>$p < 0.001$	(Aggarwal and Gupta, 1998)
Flow cytometry measurement of Bcl-2	CD8+ T cells	20-29, n=10<br>65-95, n=10	Decreased<br>$p < 0.001$	(Aggarwal and Gupta, 1998)

Flow cytometry measurement of CD95+	CD25+ T cells	<35, n=18<br>>80, n=18	No change	(Phelouzat et al., 1997)
Flow cytometry measurement of CD95+	CD3+ T cells	26.4 ± 5.1, n=34<br>73.8 ± 8.3, n=32	Increased<br>34.2-46.4 %<br>$p < 0.001$	(Schindowski et al., 2000)
Flow cytometry measurement of CD95+	CD4+ CD45RA+ T cells	20-29, n=10<br>65-95, n=10	Increase<br>6-17 %<br>$p < 0.001$	(Aggarwal and Gupta, 1998)
Flow cytometry measurement of CD95+	CD4+ CD45RO+ T cells	20-29, n=10<br>65-95, n=10	Increase<br>25-40 %<br>$p < 0.001$	(Aggarwal and Gupta, 1998)
Flow cytometry measurement of CD95+	CD4+ T cells	20-29, n=10<br>65-95, n=10	Increase<br>29-45 %<br>$p < 0.001$	(Aggarwal and Gupta, 1998)
Flow cytometry measurement of CD95+	CD4+ T cells	0, n=8<br>26.0±3, n=8<br>52.3±0.7, n=8	Increased<br>$p < 0.001$	(Shinohara et al., 1995)
Flow cytometry measurement of CD95+	CD4+ T cells	<35, n=23<br>>80, n=20	Increased<br>$p < 0.005$	(Phelouzat et al., 1997)
Flow cytometry measurement of CD95+	CD45R0+ T cells	<35, n=23<br>>80, n=20	Increased<br>$p < 0.005$	(Phelouzat et al., 1997)
Flow cytometry measurement of CD95+	CD45RA+ T cells	<35, n=20<br>>80, n=20	Increased<br>$p < 0.005$	(Phelouzat et al., 1997)
Flow cytometry measurement of CD95+	CD69+ T cells	<35, n=10<br>>80, n=10	No change	(Phelouzat et al., 1997)
Flow cytometry measurement of CD95+	CD8+ T cells	<35, n=23<br>>80, n=20	Increased<br>$p < 0.005$	(Phelouzat et al., 1997)
Flow cytometry measurement of CD95+	CD8+ CD45RA+ T cells	20-29, n=10<br>65-95, n=10	Increase<br>7-20 %<br>$p < 0.001$	(Aggarwal and Gupta, 1998)
Flow cytometry measurement of CD95+	CD8+ CD45RO+ T cells	20-29, n=10<br>65-95, n=10	Increase<br>19-35 %<br>$p < 0.001$	(Aggarwal and Gupta, 1998)

Flow cytometry measurement of CD95+	CD8+ T cells	20-29, n=10<br>65-95, n=10	Increase<br>35-56 %<br>$p < 0.001$	(Aggarwal and Gupta, 1998)
Flow cytometry measurement of CD95+	CD8+ T cells	0, n=8<br>26.0±3, n=8<br>52.3±0.7, n=8	Increased<br>$p < 0.01$	(Shinohara et al., 1995)
Flow cytometry measurement of CD95+	Dermal fibroblasts	Foreskin, n=2<br>Adult, n=2	Increased	(Jelaska and Korn, 1998)
Flow cytometry measurement of CD95+ MFI	Lymphocytes	21-60, n=40<br>66–80, n=24	Increased	(Potestio et al., 1998)
Flow cytometry measurement of CD95+	Monocytes (CD14+)	0, n=8<br>26.0±3, n=8<br>52.3±0.7, n=8	No change	(Shinohara et al., 1995)
Flow cytometry measurement of CD95+	T cells	<35<br>>80	Increased<br>$p < 0.001$	(Phelouzat et al., 1997)
Flow cytometry measurement of CD95+ after α-CD3 stimulation	T cells	<35, n=9<br>>80, n=9	Increased<br>$p < 0.05$	(Phelouzat et al., 1997)
Flow cytometry measurement of CD95+ after α-CD95 stimulation	T cells	<35, n=18<br>>80, n=15	Increased<br>$p < 0.01$	(Phelouzat et al., 1997)
Flow cytometry measurement of CD95+ after PHA stimulation	PBMCs	16-19,<br>45-55<br>n=24 total	Increase<br>$p < 0.0001$	(Li et al., 2020)
Flow cytometry measurement of CD95+ after stimulation with PHA	PBMCs	21-60, n=10<br>66–80, n=10	Increased	(Potestio et al., 1998)
Western blot measurement of Bax	Lymphocytes	20-29, n=3<br>65-95, n=3	Increased<br>$p < 0.001$	(Aggarwal and Gupta, 1998)
Western blot measurement of Bcl-2	Lymphocytes	20-29, n=3<br>65-95, n=3	Decreased<br>$p < 0.001$	(Aggarwal and Gupta, 1998)
Western blot measurement of Bcl-X_L protein	Lymphocytes	20-29, n=3<br>65-95, n=3	No change	(Aggarwal and Gupta, 1998)
Loss of proteostasis				

20S proteasomal activity	Dermal fibroblasts	45-60, n=7<br>25-35, n=3	Decrease	(Sarkar et al., 2020)
20S proteasomal activity	Endothelial cells	45-60, n=7<br>25-35, n=3	Decrease	(Sarkar et al., 2020)
Caspase-like activity of the 20S proteasome	PBMCs	40±13, n=15<br>73±6, n=21	Decrease<br>$p < 0.01$	(Papanagnou et al., 2018)
Caspase-like activity of the 20S proteasome	PBMCs	30±10, n=12<br>73±8, n=13	No change	(Papanagnou et al., 2018)
Chymotrypsin-like activity of the 20S proteasome	Dermal fibroblasts	20-78 years, n=24	Decrease ~20-50 years.<br>No change ~50-78 years	(Hwang et al., 2007)
Chymotrypsin-like activity of the 20S proteasome	PBMCs	40±13, n=15<br>73±6, n=21	Decrease<br>$p < 0.01$	(Papanagnou et al., 2018)
Chymotrypsin-like activity of the 20S proteasome	PBMCs	30±10, n=12<br>73±8, n=13	No change	(Papanagnou et al., 2018)
Expression of 11S proteasome activator mRNA by RT-qPCR	Dermal fibroblasts	68-77, n=3<br>20-30, n=3	No change	(Hwang et al., 2007)
Expression of 19S regulatory proteasome subunit mRNA by RT-qPCR	Dermal fibroblasts	68-77, n=3<br>20-30, n=3	Decrease	(Hwang et al., 2007)
Expression of 19S regulatory proteasome subunits by Western blot	Dermal fibroblasts	68-77, n=3<br>20-30, n=3	Decrease	(Hwang et al., 2007)
Expression of 20S catalytic proteasome subunit mRNA by RT-qPCR	Dermal fibroblasts	68-77, n=3<br>20-30, n=3	Decrease	(Hwang et al., 2007)
Expression of 20S catalytic proteasome subunits by Western blot	Dermal fibroblasts	68-77, n=3<br>20-30, n=3	Decrease	(Hwang et al., 2007)
Expression of the 11S proteasome activator PSME1 by Western blot	Dermal fibroblasts	68-77, n=3<br>20-30, n=3	Increase	(Hwang et al., 2007)
Peptidylglutamyl–peptide hydrolase-like activity of the 20S proteasome	Dermal fibroblasts	20-78 years, n=36	Decrease ~20-50 years<br>No change ~50-78 years	(Hwang et al., 2007)
Protein oxidation by protein–carbonyl Western blot	Dermal fibroblasts	68-77, n=3<br>20-30, n=3	Increase	(Hwang et al., 2007)

Protein oxidation by protein–carbonyl Western blot	PBMCs	24-26, n=2<br>80-89, n=3	Increased	(Papanagnou et al., 2018)
Protein ubiquitination by Western blot	Dermal fibroblasts	68-77, n=3<br>20-30, n=3	Increase	(Hwang et al., 2007)
Protein ubiquitination by Western blot	PBMCs	24-26, n=2<br>80-89, n=3	Increased	(Papanagnou et al., 2018)
Trypsin-like activity of the 20S proteasome	Dermal fibroblasts	20-78 years, n=36	Decrease ~20-50 years<br>No change ~50-78 years	(Hwang et al., 2007)
Trypsin-like activity of the 20S proteasome	PBMCs	40±13, n=15<br>73±6, n=21	Decrease<br>$p < 0.01$	(Papanagnou et al., 2018)
Trypsin-like activity of the 20S proteasome	PBMCs	30±10, n=12<br>73±8, n=13	Decrease<br>$p < 0.05$	(Papanagnou et al., 2018)
Macro-autophagy				
Autophagosome fluorescent staining	Dermal fibroblasts	45-60, n=7<br>25-35, n=3	Decrease	(Sarkar et al., 2020)
Autophagosome fluorescent staining	Endothelial cells	45-60, n=7<br>25-35, n=3	Decrease	(Sarkar et al., 2020)
Intercellular communication				
Ca^{2+} signalling after stimulation with PHA by flow cytometry with Fluo-3 stain.	T cells	21-60, n=3<br>66–80, n=3	Decreased<br>$p < 0.05$	(Potestio et al., 1998)
CD25 by flow cytometry (mean fluorescence intensity)	T cells	30±5, n=21<br>85±8, n=35	No change	(Serra et al., 1996)
CD25 by flow cytometry (mean fluorescence intensity) after phorbol myristate acetate stimulation	T cells	30±5, n=21<br>85±8, n=35	No change	(Serra et al., 1996)
CD25 by flow cytometry (mean fluorescence intensity) after phorbol myristate acetate and α-CD69 stimulation	T cells	30±5, n=21<br>85±8, n=35	Decreased	(Serra et al., 1996)
CD28 expression after PHA stimulation	T cells	16–19<br>45-55<br>n=24 total	Decrease<br>$p < 0.01$	(Li et al., 2020)
CD3 expression after PHA stimulation	T cells	16–19<br>45-55	No change	(Li et al., 2020)

		n=24 total		
GROα (CXCL1) secretion	Endothelial cells	25-35, n=3<br>45-60, n=7	Increase	(Sarkar et al., 2020)
HLA-DR expression by flow cytometry	CD3+ T cells	26.4±5.1, n=34)<br>73.8±8.3, n=32	Increased<br>$p < 0.0001$	(Schindowski et al., 2000)
IL-10 secretion after influenza stimulation measured by cytometric bead assay	PBMCs	25-46, n=7<br>81-98, n=13	Decreased<br>63%<br>$p < 0.03$	(Deng et al., 2004)
IL-10 secretion after stimulation with LPS or PTX3+LPS	PBMCs	25 ± 3, n=15<br>57 ± 7, n=15	No change	(Slusher et al., 2019)
IL-10 secretion after stimulation with PTX3	PBMCs	25 ± 3, n=15<br>57 ± 7, n=15	Decrease<br>$p = 0.016$	(Slusher et al., 2019)
IL-18 secretion	Endothelial cells	25-35, n=3<br>45-60, n=7	Increase	(Sarkar et al., 2020)
IL-1A secretion	Endothelial cells	25-35, n=3<br>45-60, n=7	Increase	(Sarkar et al., 2020)
IL-1β secretion by ELISA after PHA stimulation	PBMCs	20 – 40, n=26<br>>60, n=44	No change	(Ahluwalia et al., 2001)
IL-2 expression by qPCR	PBMCs	16–19<br>45-55 years<br>n=24 total	Decrease<br>$p < 0.0001$	(Li et al., 2020)
IL-2 receptor expression by flow cytometry	CD3+ T cells	26.4±5.1, n=34)<br>73.8±8.3, n=32	No change	(Schindowski et al., 2000)
IL-2 secretion after influenza stimulation measured by cytometric bead assay	PBMCs	25-46, n=7<br>81-98, n=13	No change	(Deng et al., 2004)
IL-2 secretion after stimulation with concanavalin A measured by ELISA	PBMCs	23.35±3.21, n=5<br>73.12±5.36, n=5	Decreased<br>$p < 0.05$	(Bouamama et al., 2021)
IL-2 secretion by ELISA	PBMCs	16–19<br>45-55 years<br>n=24 total	Decrease<br>$p < 0.01$	(Li et al., 2020)
IL-2 secretion by ELISA after PHA stimulation	PBMCs	20 – 40, n=26<br>>60, n=44	No change	(Ahluwalia et al., 2001)

IL-22 secretion	Endothelial cells	25-35, n=3<br>45-60, n=7	No change	(Sarkar et al., 2020)
IL-4 secretion after stimulation with concanavalin A measured by ELISA	PBMCs	23.35±3.21, n=5<br>73.12±5.36, n=5	Increased<br>$p < 0.05$	(Bouamama et al., 2021)
IL-4 secretion after stimulation with PHA measured by ELISA	PBMCs	20-87, n=138	No change	(Candore et al., 1993)
IL-6 mRNA by qPCR	CD8+ T cells	23-30, n=18<br>57-67, n=16	No change	(Martínez-Zamudio et al., 2021)
IL-6 secretion after cytomegalovirus exposure measured by ELISA	Dermal fibroblasts	21-26, n=5<br>90-92, n=8	Increased<br>$p < 0.05$	(Wolf et al., 2012)
IL-6 secretion after LPS stimulation measured by ELISA	Dermal fibroblasts	21-26, n=5<br>90-92, n=8	Increased<br>$p < 0.05$	(Wolf et al., 2012)
IL-6 secretion after stimulation with concanavalin A measured by ELISA	PBMCs	23.35±3.21, n=5<br>73.12±5.36, n=5	Decreased<br>$p < 0.05$	(Bouamama et al., 2021)
IL-6 secretion after stimulation with Concanavalin A with autologous plasma measured by ELISA	PBMCs	20–30, n=21<br>>65, n=26	Decreased<br>$p < 0.05$	(Beharka et al., 2001)
IL-6 secretion after stimulation with Concanavalin A with FBS measured by ELISA	PBMCs	20–30, n=21<br>>65, n=26	No change	(Beharka et al., 2001)
IL-6 secretion after stimulation with LPS	PBMCs	25 ± 3, n=15<br>57 ± 7, n=15	No change	(Slusher et al., 2019)
IL-6 secretion after stimulation with PHA cultured in RPMI with autologous plasma measured by ELISA	PBMCs	20–30, n=21<br>>65 years, n=26	No change	(Beharka et al., 2001)
IL-6 secretion after stimulation with PHA cultured in RPMI with FBS measured by ELISA	PBMCs	20–30, n=21<br>>65, n=26	No change	(Beharka et al., 2001)
IL-6 secretion after stimulation with PHA measured by ELISA	PBMCs	20-87, n=138	No change	(Candore et al., 1993)
IL-6 secretion after stimulation with PTX3 or PTX3+LPS	PBMCs	25 ± 3, n=15<br>57 ± 7, n=15	Increase<br>$p = 0.009$	(Slusher et al., 2019)
IL-6 secretion by ELISA after PHA stimulation	PBMCs	20 – 40, n=26<br>>60, n=44	No change	(Ahluwalia et al., 2001)

IL-6 secretion by single-cell secretion microchip technology	Dermal fibroblast	3-92 years, n=9	Increased<br>R = 0.52	(Phillip et al., 2017)
IL-6 secretion measured by ELISA	Dermal fibroblasts	21-26, n=5<br>90-92, n=8	Increased<br>$p < 0.05$	(Wolf et al., 2012)
IL-6 secretion measured by ELISA	PBMCs	20–30, n=21<br>>65, n=26	No change	(Beharka et al., 2001)
IL-8 secretion	Endothelial cells	25-35, n=3<br>45-60, n=7	Increase	(Sarkar et al., 2020)
IL-8 secretion after cytomegalovirus exposure measured by ELISA	Dermal fibroblasts	21-26, n=5<br>90-92, n=8	Increased<br>$p < 0.05$	(Wolf et al., 2012)
IL-8 secretion after LPS stimulation measured by ELISA	Dermal fibroblasts	21-26, n=5<br>90-92, n=8	Increased<br>$p < 0.05$	(Wolf et al., 2012)
IL-8 secretion measured by ELISA	Dermal fibroblasts	21-26, n=5<br>90-92, n=8	Increased<br>$p < 0.05$	(Wolf et al., 2012)
IL-9 secretion	Endothelial cells	25-35, n=3<br>45-60, n=7	Increase	(Sarkar et al., 2020)
INF-γ expression by qPCR	PBMCs	16–19<br>45-55 years<br>n=24 total	Decrease<br>$p < 0.0001$	(Li et al., 2020)
INF-γ mean fluorescent intensity after influenza stimulation measured by flow cytometry.	INF-γ and CD69+ T cells	24-38, n=5<br>70-94, n=11	Decreased 42 %<br>$p = 0.04$	(Deng et al., 2004)
INF-γ positive cells after stimulation with influenza virus	PBMCs	21- 35, n=6<br>67-88, n=18	Decreased<br>$p < 0.03$	(Deng et al., 2004)
INF-γ positive cells after stimulation with varicella zoster virus	PBMCs	21- 35, n=6<br>67-88, n=18	Decreased<br>$p < 0.01$	(Deng et al., 2004)
INF-γ secretion after influenza stimulation measured by cytometric bead assay	PBMCs	(25- 46, n=7<br>81-98, n=13	Decreased 70-81%<br>$p < 0.03$	(Deng et al., 2004)
INF-γ secretion after stimulation with PHA	PBMCs	20-87, n=138	Decreased<br>R = -0.24<br>$p = 0.016$	(Candore et al., 1993)
INF-γ secretion by ELISA	PBMCs	16–19	Decrease	(Li et al., 2020)

		45-55 years<br>n=24 total	$p < 0.01$	
Intracellular glutathione levels after concanavalin A stimulation	PBMCs	23.35±3.21, n=5<br>73.12±5.36, n=5	No change	(Bouamama et al., 2021)
Nitric oxide secretion after stimulation with concanavalin A	PBMCs	23.35±3.21, n=5<br>73.12±5.36, n=5	Decreased<br>$p = 0.02$	(Bouamama et al., 2021)
T cell activation (CD69+ expression) after stimulation with α-CD3 by flow cytometry.	Rosetting T cells	30±5, n=21<br>85±8, n=35	Decreased<br>79.5-28.1 %<br>$p < 0.0002$	(Serra et al., 1996)
T cell activation (CD69+ expression) after stimulation with phorbol myristate acetate by flow cytometry.	Rosetting T cells	30±5, n=21<br>85±8, n=35	No change	(Serra et al., 1996)
T cell activation (CD69+ expression) by flow cytometry.	Rosetting T cells	30±5, n=21<br>85±8, n=35	Decreased<br>10-3.4 %<br>$p < 0.0003$	(Serra et al., 1996)
TGF-β secretion after stimulation with LPS, PTX3 or PTX3+LPS	PBMCs	25 ± 3, n=15<br>57 ± 7, n=15	No change	(Slusher et al., 2019)
TNF-β expression by qPCR	PBMCs	16–19<br>45-55 years<br>n=24 total	Increase<br>$p < 0.05$	(Li et al., 2020)
TNF-α secretion after influenza stimulation measured by cytometric bead assay	PBMCs	25-46, n=7<br>81-98, n=13	Decreased	(Deng et al., 2004)
TNF-β secretion by ELISA	PBMCs	16–19<br>45-55 years<br>n=24 total	Increase<br>$p < 0.001$	(Li et al., 2020)
TNF-α secretion after stimulation with LPS, PTX3 or PTX3+LPS	PBMCs	25 ± 3, n=15<br>57 ± 7, n=15	No change	(Slusher et al., 2019)
Extracellular matrix changes				
Collagen COL1A2 mRNA expression	Dermal fibroblast	24.8±8.3, n=5<br>59.8 ± 3.7, n = 5<br>74.4 ± 2.3, n = 5	No change	(Rorteau et al., 2022)
Collagen deposition	Dermal fibroblast	24.8±8.3, n=5<br>59.8 ± 3.7, n = 5<br>74.4 ± 2.3, n = 5	Decreased 38 %<br>$p < 0.01$	(Rorteau et al., 2022)

Elastin deposition	Dermal fibroblast	24.8±8.3, n=5<br>59.8 ± 3.7, n = 5<br>74.4 ± 2.3, n = 5	Decreased 2-fold in old<br>$p < 0.01$	(Rorteau et al., 2022)
Fibronectin deposition	Dermal fibroblast	24.8±8.3, n=5<br>59.8 ± 3.7, n = 5<br>74.4 ± 2.3, n = 5	Decreased 18 %<br>$p < 0.05$	(Rorteau et al., 2022)
Integrin-α6+ basal cells by fluorescence microscopy	Keratinocytes	18-90, n=52	Decreased	(Rübe et al., 2021)
Matrix metalloproteinase 1 (MMP1) mRNA expression	Dermal fibroblast	24.8±8.3, n=5<br>59.8 ± 3.7, n = 5<br>74.4 ± 2.3, n = 5	No change	(Rorteau et al., 2022)
Matrix metalloproteinase 3 (MMP3) mRNA expression	Dermal fibroblast	24.8±8.3, n=5<br>59.8 ± 3.7, n = 5<br>74.4 ± 2.3, n = 5	No change	(Rorteau et al., 2022)
Matrix metalloproteinase 12 (MMP12) activity	Dermal fibroblast	24.8±8.3, n=5<br>59.8 ± 3.7, n = 5<br>74.4 ± 2.3, n = 5	Decreased 25 % in middle aged and 50 % in elderly<br>$p < 0.001$	(Rorteau et al., 2022)
Matrix metalloproteinase 12 (MMP12) mRNA expression	Dermal fibroblast	24.8±8.3, n=5<br>59.8 ± 3.7, n = 5<br>74.4 ± 2.3, n = 5	No change	(Rorteau et al., 2022)
Mitochondrial dysfunction				
ATP linked respiration measured by Seahorse assay	Dermal fibroblast	24.8±8.3, n=5<br>59.8±3.7, n=5<br>74.4±2.3, n=5	Decreased in old<br>$p < 0.0001$	(Rorteau et al., 2022)
ATP production	Dermal fibroblast	2 – 92 years, n=9	Decreased<br>R = -0.64	(Phillip et al., 2017)
Basal oxygen consumption rate measured by Seahorse assay	Dermal fibroblast	24.8±8.3, n=5<br>59.8±3.7, n=5<br>74.4±2.3, n=5	Increased<br>Young and middle aged 40 pmol/min<br>Aged 67 pmol/min	(Rorteau et al., 2022)

			$p < 0.0001$	
DHR 123 staining for reactive oxygen species	Lymphocytes	26.4±5.1, n=34<br>73.8±8.3, n=32	Increased<br>$p < 0.05$	(Schindowski et al., 2000)
MitoSOX staining for mitochondrial superoxide	Dermal fibroblasts	60-70, n=8<br>25-35, n=3	Increase	(Sarkar et al., 2020)
MitoSOX staining for mitochondrial superoxide	Dermal fibroblasts	11, n=3<br>71-83, n=3	Increase	(Miller et al., 2013)
MitoSOX staining for mitochondrial superoxide	Endothelial cells	45-60, n=7<br>25-35, n=3	Increase	(Sarkar et al., 2020)
mRNA levels of oxidation stress response gene CAT	Dermal fibroblast	24.8±8.3, n=5<br>59.8±3.7, n=5<br>74.4±2.3, n=5	No change	(Rorteau et al., 2022)
mRNA levels of oxidation stress response gene HMOX1	Dermal fibroblast	24.8±8.3, n=5<br>59.8±3.7, n=5<br>74.4±2.3, n=5	No change	(Rorteau et al., 2022)
mRNA levels of oxidation stress response gene SOD2	Dermal fibroblast	24.8±8.3, n=5<br>59.8±3.7, n=5<br>74.4±2.3, n=5	No change	(Rorteau et al., 2022)
mtDNA copies per cell	Dermal fibroblast	24.8±8.3, n=5<br>59.8±3.7, n=5<br>74.4±2.3, n=5	No change	(Rorteau et al., 2022)
Non-mitochondria respiration measured by Seahorse assay	Dermal fibroblast	24.8±8.3, n=5<br>59.8±3.7, n=5<br>74.4±2.3, n=5	Increased in middle aged and old<br>$p < 0.0001$	(Rorteau et al., 2022)
Proton leak measured by Seahorse assay	Dermal fibroblast	24.8±8.3, n=5<br>59.8±3.7, n=5<br>74.4±2.3, n=5	Increased in old<br>$p < 0.0001$	(Rorteau et al., 2022)
Respiration reserve capacity measured by Seahorse assay	Dermal fibroblast	24.8±8.3, n=5<br>59.8±3.7, n=5<br>74.4±2.3, n=5	Decreased in middle aged and old<br>$p < 0.0001$	(Rorteau et al., 2022)

Surtuin1 immunocytochemistry	Dermal fibroblasts	60-70, n=8<br>25-35, n=3	Decrease	(Sarkar et al., 2020)
Surtuin1 immunocytochemistry	Endothelial cells	45-60, n=7<br>25-35, n=3	Decrease	(Sarkar et al., 2020)
Tetramethylrhodamine Methyl Ester Perchlorate staining for mitochondrial membrane potential	Dermal fibroblasts	60-70, n=8<br>25-35, n=3	Decrease	(Sarkar et al., 2020)
Tetramethylrhodamine Methyl Ester Perchlorate staining for mitochondrial membrane potential	Endothelial cells	45-60, n=7<br>25-35, n=3	Decrease	(Sarkar et al., 2020)
Senescence				
β-galactosidase staining	Dermal fibroblasts	25-35, n=3<br>60-70, n=8	No change	(Sarkar et al., 2020)
β-galactosidase staining	Dermal fibroblasts	20-30, n=3<br>68-77, n=3	Increased	(Hwang et al., 2007)
β-galactosidase staining	Dermal fibroblasts	24.8±8.3, n=5<br>59.8 ± 3.7, n=5<br>74.4±2.3, n=5	No change	(Rorteau et al., 2022)
β-galactosidase staining	Endothelial cells	25-35, n=3<br>45-60, n=7	Increased	(Sarkar et al., 2020)
β-galactosidase staining after treatment with etoposide	Dermal fibroblasts	24.8±8.3, n=5<br>59.8 ± 3.7, n=5<br>74.4±2.3, n=5	Increased<br>31.6% ><br>44.8% ><br>45.6%	(Rorteau et al., 2022)
53BP1 immunostaining	Dermal fibroblasts	24.8 ± 8.3, n=5<br>59.8 ± 3.7, n=5<br>74.4 ± 2.3, n=5	No change	(Rorteau et al., 2022)
BP53 levels by fluorescence microscopy	Keratinocytes	<30, n=3<br>30-60, n=3<br>>60, n-3	Increased<br>p < 0.001	(Rübe et al., 2021)
Ki67 levels by fluorescence microscopy	Keratinocytes	<30, n=3<br>30-60, n=3<br>>60, n-3	Decreased<br>p < 0.001	(Rübe et al., 2021)
P16 mRNA expression	Dermal fibroblasts	24.8±8.3, n=5<br>59.8 ± 3.7, n=5	No change	(Rorteau et al., 2022)

		74.4±2.3, n=5		
P16 protein expression by Western blot	Dermal fibroblasts	21-26, n=5<br>90-92, n=8	No change	(Wolf et al., 2012)
p16^{INK4a} mRNA by qPCR	CD8+ T cells	23-30, n=18<br>57-67, n=16	No change	(Martínez-Zamudio et al., 2021)
P21 mRNA by qPCR	CD8+ T cells	23-30, n=18<br>57-67, n=16	No change	(Martínez-Zamudio et al., 2021)
P21 protein levels (Western blot)	Dermal fibroblasts	20-30, n=3<br>68-77, n=3	Increased	(Hwang et al., 2007)
Proliferation after Concanavalin A stimulation	PBMCs	23.35±3.21, n=5<br>73.12±5.36, n=5	Decreased<br>$p = 0.04$	(Bouamama et al., 2021)
Proliferation after polyclonal stimulation measured by cell trace violet	Central memory T cells	19–30, n=5,<br>60-70, n=5	No change	(Quinn et al., 2018)
Proliferation after polyclonal stimulation measured by cell trace violet	Effector memory T cells	19–30, n=5,<br>60-70, n=5	No change	(Quinn et al., 2018)
Proliferation after polyclonal stimulation measured by cell trace violet	Naiive T cells	19–30, n=5,<br>60-70, n=5	No change	(Quinn et al., 2018)
Proliferation after polyclonal stimulation measured by cell trace violet	Terminally differentiated effector memory T cells	19–30, n=5,<br>60-70, n=5	No change	(Quinn et al., 2018)
Proliferation after polyclonal stimulation measured by cell trace violet	Virtual memory T cells	19–30, n=5,<br>60-70, n=5	Decreased<br>$p < 0.05$	(Quinn et al., 2018)
Proliferation by [^{3}H]TdR incorporation	PBMCs	30±5, n=21<br>85±8, n=35	No change	(Serra et al., 1996)
Proliferation by [^{3}H]TdR incorporation after α-CD69 and α-CD3 stimulation	PBMCs	30±5, n=21<br>85±8, n=35	Decreased<br>$p < 0.02$	(Serra et al., 1996)
Proliferation by [^{3}H]TdR incorporation after α-CD69 and phorbol myristate acetate stimulation	PBMCs	30±5, n=21<br>85±8, n=35	Decreased<br>$p < 0.02$	(Serra et al., 1996)

Proliferation by $[^{3}H]$TdR incorporation after α-CD69 stimulation	PBMCs	30±5, n=21<br>85±8, n=35	No change	(Serra et al., 1996)
Proliferation by $[^{3}H]$TdR incorporation after and α-CD3 stimulation	PBMCs	30±5, n=21<br>85±8, n=35	Decreased<br>$p < 0.02$	(Serra et al., 1996)
Proliferation by $[^{3}H]$TdR incorporation after and phorbol myristate acetate stimulation	PBMCs	30±5, n=21<br>85±8, n=35	No change	(Serra et al., 1996)
Proliferation by $[^{3}H]$TdR incorporation after IL-2 and α-CD3 stimulation	PBMCs	30±5, n=21<br>85±8, n=35	Decreased<br>$p < 0.03$	(Serra et al., 1996)
Proliferation by $[^{3}H]$TdR incorporation after IL-2 and α-CD69 stimulation	PBMCs	30±5, n=21<br>85±8, n=35	Decreased<br>$p < 0.001$	(Serra et al., 1996)
Proliferation by $[^{3}H]$TdR incorporation after IL-2 and phorbol myristate acetate stimulation	PBMCs	30±5, n=21<br>85±8, n=35	No change	(Serra et al., 1996)
Proliferation by $[^{3}H]$TdR incorporation after IL-2 stimulation	PBMCs	30±5, n=21<br>85±8, n=35	Decreased<br>$p < 0.001$	(Serra et al., 1996)
Proliferation by $[^{3}H]$TdR incorporation after IL-2, α-CD69 and α-CD3 stimulation	PBMCs	30±5, n=21<br>85±8, n=35	Decreased<br>$p < 0.003$	(Serra et al., 1996)
Proliferation by $[^{3}H]$TdR incorporation after IL-2, α-CD69 and phorbol myristate acetate stimulation	PBMCs	30±5, n=21<br>85±8, n=35	Decreased<br>$p < 0.005$	(Serra et al., 1996)
Proliferation in culture	NK cells	41-50<br>51-60<br>61-70<br>71-80<br>n=40 total	Decreased	(Gounder et al., 2018)
Proliferation measured by EdU incorporation	T cells	16-19,<br>45-55<br>n=24 total	Decreased<br>$p < 0.05$	(Li et al., 2020)
Proliferation measured by EdU incorporation after PHA stimulation	T cells	16-19,<br>45-55<br>n=24 total	Decreased<br>$p < 0.0001$	(Li et al., 2020)
Proliferation rate after stimulation with IL-2	PBMCs	21-60, n=3<br>66–80, n=3	Decreased<br>$p < 0.05$	(Potestio et al., 1998)
Proliferative potential	Dermal fibroblasts	24.8±8.3, n=5<br>59.8 ± 3.7, n=5	Decreased	(Rorteau et al., 2022)

		74.4±2.3, n=5	14 > 10 > 4 population doublings	
Proportion of proliferating cells	CD8+ T cells	23-30, n=18<br>57-67, n=16	No change	(Martínez-Zamudio et al., 2021)
Proportion of β-galactosidase positive cells by flow cytometry	B cells	23-30, n=18<br>57-67, n=16	No change	(Martínez-Zamudio et al., 2021)
Proportion of β-galactosidase positive cells by flow cytometry	CD4+ T cells	23-30, n=18<br>57-67, n=16	Increased<br>$p < 0.05$	(Martínez-Zamudio et al., 2021)
Proportion of β-galactosidase positive cells by flow cytometry	CD8+ T cells	23-30, n=18<br>57-67, n=16	Increased<br>$p < 0.0001$	(Martínez-Zamudio et al., 2021)
Proportion of β-galactosidase positive cells by flow cytometry	Central memory CD8+ T cells	23-30, n=8<br>57-67, n=8	No change	(Martínez-Zamudio et al., 2021)
Proportion of β-galactosidase positive cells by flow cytometry	Effector memory CD8+ T cells	23-30, n=8<br>57-67, n=8	No change	(Martínez-Zamudio et al., 2021)
Proportion of β-galactosidase positive cells by flow cytometry	Monocytes	23-30, n=18<br>57-67, n=16	No change	(Martínez-Zamudio et al., 2021)
Proportion of β-galactosidase positive cells by flow cytometry	Naïve CD8+ T cells	23-30, n=8<br>57-67, n=8	No change	(Martínez-Zamudio et al., 2021)
Proportion of β-galactosidase positive cells by flow cytometry	Natural killer cells	23-30, n=18<br>57-67, n=16	No change	(Martínez-Zamudio et al., 2021)

Proportion of β-galactosidase positive cells by flow cytometry	Plasmacytoid dendrocytes	23-30, n=18<br>57-67, n=16	No change	(Martínez-Zamudio et al., 2021)
Proportion of β-galactosidase positive cells by flow cytometry	Terminally differentiated effector memory CD8+ T cells	23-30, n=8<br>57-67, n=8	Increased<br>$p = 0.047$	(Martínez-Zamudio et al., 2021)
Changes in cell populations				
B cell receptor diversity	B cells	20-45, n=8<br>60-80, n=8	No change	(Zheng et al., 2020)
B cells receptor diversity	Age associated B cells	20-45, n=8<br>60-80, n=8	No change	(Zheng et al., 2020)
B cells receptor diversity	Antibody secreting B cells	20-45, n=8<br>60-80, n=8	No change	(Zheng et al., 2020)
B cells receptor diversity	Memory B cells	20-45, n=8<br>60-80, n=8	No change	(Zheng et al., 2020)
B cells receptor diversity	Naïve B cells	20-45, n=8<br>60-80, n=8	No change	(Zheng et al., 2020)
CD16+ T cells as a proportion of lymphocytes by flow cytometry	Lymphocytes	21-60, n=40<br>66–80, n=24	No change	(Potestio et al., 1998)
CD19+ T cells as a proportion of lymphocytes by flow cytometry	Lymphocytes	21-60, n=40<br>66–80, n=24	No change	(Potestio et al., 1998)
CD3+ T cells as a proportion of lymphocytes by flow cytometry	Lymphocytes	21-60, n=40<br>66–80, n=24	No change	(Potestio et al., 1998)
CD4+ T cells as a proportion of lymphocytes by flow cytometry	Lymphocytes	21-60, n=40<br>66–80, n=24	No change	(Potestio et al., 1998)
CD8+ T cells as a proportion of lymphocytes by flow cytometry	Lymphocytes	21-60, n=40<br>66–80, n=24	No change	(Potestio et al., 1998)
IFN-γ and CD69+ T cells after influenza stimulation measured by flow cytometry	PBMCs	24-38, n=5<br>70-94, n=11	Decreased by 48 %<br>$p = 0.01$	(Deng et al., 2004)

Proportion of CD14+ monocytes measured by mass cytometry	PBMCs	20-45, n=8<br>60-80, n=8	Increased<br>$p = 0.0012$	(Zheng et al., 2020)
Proportion of CD14+ monocytes measured by ssRNA-seq	PBMCs	20-45, n=8<br>60-80, n=8	Increased<br>$p = 0.0006$	(Zheng et al., 2020)
Proportion of CD4- CD8- T cells measured by flow cytometry	PBMCs	24-38, n=5<br>70-94, n=11	No change	(Deng et al., 2004)
Proportion of CD4+ naïve T cells measured by mass cytometry	PBMCs	20-45, n=8<br>60-80, n=8	Decreased<br>$p = 0.0175$	(Zheng et al., 2020)
Proportion of CD4+ naïve T cells measured by ssRNA-seq	PBMCs	20-45, n=8<br>60-80, n=8	Decreased<br>$p = 0.0279$	(Zheng et al., 2020)
Proportion of CD4+ T cells measured by flow cytometry	PBMCs	24-38, n=5<br>70-94, n=11	No change	(Deng et al., 2004)
Proportion of CD8+ naïve T cells measured by mass cytometry	PBMCs	20-45, n=8<br>60-80, n=8	No change	(Zheng et al., 2020)
Proportion of CD8+ naïve T cells measured by ssRNA-seq	PBMCs	20-45, n=8<br>60-80, n=8	Decreased<br>$p = 0.0005$	(Zheng et al., 2020)
Proportion of CD8+ T cells measured by flow cytometry	PBMCs	24-38, n=5<br>70-94, n=11	No change	(Deng et al., 2004)
Proportion of central memory T cells ($CD45RA^{lo}CD27^{hi}$) measured by flow cytometry	CD8+ T cells	19–30, n=5,<br>60-70, n=5	No change	(Quinn et al., 2018)
Proportion of central memory T cells measured by flow cytometry	CD8+ T cells	23-30, n=8<br>57-67, n=8	No change	(Martínez-Zamudio et al., 2021)
Proportion of effector memory T cells ($CD45RA^{lo}CD27^{lo}$) measured by flow cytometry	CD8+ T cells	19–30, n=5,<br>60-70, n=5	No change	(Quinn et al., 2018)
Proportion of effector memory T cells measured by flow cytometry	CD8+ T cells	23-30, n=8<br>57-67, n=8	Increased	(Martínez-Zamudio et al., 2021)
Proportion of naïve T cells ($CD45RA^{hi}CD27^{hi}PanNK^{-}NKG2A^{-}$) measured by flow cytometry	CD8+ T cells	19–30, n=5,<br>60-70, n=5	Decreased<br>$p < 0.05$	(Quinn et al., 2018)

Proportion of naïve T cells measured by flow cytometry	CD8+ T cells	23-30, n=8<br>57-67, n=8	Decreased	(Martínez-Zamudio et al., 2021)
Proportion of NK cells	Lymphocytes	41-50<br>51-60<br>61-70<br>71-80<br>n=40 total	Increased	(Gounder et al., 2018)
Proportion of T memory cells by flow cytometry measurement of CD45RO+	CD4+ T cells	0, n=8<br>26.0±3, n=8<br>52.3±0.7, n=8	Increased	(Shinohara et al., 1995)
Proportion of T memory cells by flow cytometry measurement of CD45RO+	CD8+ T cells	0, n=8<br>26.0±3, n=8<br>52.3±0.7, n=8	No change	(Shinohara et al., 1995)
Proportion of terminally differentiated effector memory T cells ($CD45RA^{hi}CD27^{lo}PanNK^{-}NKG2A^{-}$) measured by flow cytometry	CD8+ T cells	19–30, n=5,<br>60-70, n=5	Increased<br>$p < 0.05$	(Quinn et al., 2018)
Proportion of terminally differentiated effector memory T cells measured by flow cytometry	CD8+ T cells	23-30, n=8<br>57-67, n=8	Increased	(Martínez-Zamudio et al., 2021)
Proportion of the most expanded B cell receptor clone	B cells	20-45, n=8<br>60-80, n=8	Increased<br>$p = 0.0305$	(Zheng et al., 2020)
Proportion of the most expanded T cell receptor clone	T cells	20-45, n=8<br>60-80, n=8	No change	(Zheng et al., 2020)
Proportion of virtual memory T cells ($CD45RA^{+}PanKIR^{+}$ and/or $NKG2A^{+}$) measured by flow cytometry	CD8+ T cells	19–30, n=5,<br>60-70, n=5	Increased<br>$p < 0.05$	(Quinn et al., 2018)
T cell receptor diversity	CD4+ central memory T cells	20-45, n=8<br>60-80, n=8	No change	(Zheng et al., 2020)
T cell receptor diversity	CD4+ effector memory T cells	20-45, n=8<br>60-80, n=8	No change	(Zheng et al., 2020)
T cell receptor diversity	CD4+ exhausted T cells	20-45, n=8<br>60-80, n=8	No change	(Zheng et al., 2020)
T cell receptor diversity	CD4+ naïve T cells	20-45, n=8<br>60-80, n=8	No change	(Zheng et al., 2020)

T cell receptor diversity	CD4+ T regulatory cells	20-45, n=8<br>60-80, n=8	No change	(Zheng et al., 2020)
T cell receptor diversity	CD4+CD8+ T cells	20-45, n=8<br>60-80, n=8	No change	(Zheng et al., 2020)
T cell receptor diversity	CD4-CD8- T cells	20-45, n=8<br>60-80, n=8	No change	(Zheng et al., 2020)
T cell receptor diversity	CD8+ cytotoxic T cells	20-45, n=8<br>60-80, n=8	Decrease<br>p = 0.0382	(Zheng et al., 2020)
T cell receptor diversity	CD8+ effector memory T cells	20-45, n=8<br>60-80, n=8	Decrease<br>p = 0.0084	(Zheng et al., 2020)
T cell receptor diversity	CD8+ exhausted T cells	20-45, n=8<br>60-80, n=8	Decrease<br>p = 0.0182	(Zheng et al., 2020)
T cell receptor diversity	CD8+ naïve T cells	20-45, n=8<br>60-80, n=8	No change	(Zheng et al., 2020)
T cell receptor diversity	Mitotic T cells	20-45, n=8<br>60-80, n=8	No change	(Zheng et al., 2020)
T cell receptor diversity	T cells	20-45, n=8<br>60-80, n=8	Decreased<br>p = 0.0379	(Zheng et al., 2020)
Other				
Cell size	Dermal fibroblast	2-92, n=9	Increased<br>R = 0.6	(Phillip et al., 2017)
Cell traction strength measured by matrix displacement	Dermal fibroblast	2-92, n=9	Increased<br>R = 0.35	(Phillip et al., 2017)
Cell traction stress anisotropy	Dermal fibroblast	2-92, n=9	Increased<br>R = 0.72	(Phillip et al., 2017)
Cytoplasmic viscosity measured by nanoparticle displacement in 1 s	Dermal fibroblast	2-92, n=9	Decreased<br>R = -0.32	(Phillip et al., 2017)
Cytoplasmic viscosity measured by nanoparticle displacement in 10 s	Dermal fibroblast	2-92, n=9	Decreased<br>R = -0.45	(Phillip et al., 2017)
Cytoskeleton changes measured by F-actin content	Dermal fibroblast	2-92, n=9	Increased<br>R = 0.33	(Phillip et al., 2017)

Cytoskeleton changes measured by F-actin fibre bundling	Dermal fibroblast	2-92, n=9	Increased<br>R = 0.38	(Phillip et al., 2017)
Lipid peroxidation levels after concanavalin A stimulation measured by malondialdehydes	PBMCs	23.35±3.21, n=5<br>73.12±5.36, n=5	Increased<br>p = 0.009	(Bouamama et al., 2021)
Scratch wound closure rate	Dermal fibroblast	2-92, n=9	Decreased<br>R = -0.73	(Phillip et al., 2017)
Scratch wound half life	Dermal fibroblast	2-92, n=9	Increased<br>R = 0.69	(Phillip et al., 2017)
Single cell motility	Dermal fibroblast	2-92, n=9	Decreased<br>R = -0.97	(Phillip et al., 2017)
Single cell motility persistence	Dermal fibroblast	2-92, n=9	Decreased<br>R = -0.86	(Phillip et al., 2017)
SIRT-1 immunocytochemistry	Vascular endothelial cells	18-30, n=16<br>59-76, n=22	Decrease	(Donato et al., 2011)

3 Supplementary references

Aggarwal, S., and Gupta, S. (1998). Increased Apoptosis of T Cell Subsets in Aging Humans: Altered Expression of Fas (CD95), Fas Ligand, Bcl-2, and Bax. *J. Immunol.* 160, 1627–1637. doi: 10.4049/jimmunol.160.4.1627.

Ahluwalia, N., Mastro, A. M., Ball, R., Miles, M. P., Rajendra, R., and Handte, G. (2001). Cytokine production by stimulated mononuclear cells did not change with aging in apparently healthy, well-nourished women. *Mech. Ageing Dev.* 122, 1269–1279. doi: 10.1016/S0047-6374(01)00266-4.

Beharka, A. A., Meydani, M., Wu, D., Leka, L. S., Meydani, A., and Meydani, S. N. (2001). Interleukin-6 production does not increase with age. *J. Gerontol. Ser. A* 56, B81–B88. doi: 10.1093/gerona/56.2.B81.

Bouamama, S., Merzouk, H., Latrech, H., Charif, N., and Bouamama, A. (2021). Royal jelly alleviates the detrimental effects of aging on immune functions by enhancing the in vitro cellular proliferation, cytokines, and nitric oxide release in aged human PBMCS. *J. Food Biochem.* 45, e13619. doi: 10.1111/jfbc.13619.

Candore, G., Lorenzo, G. D., Melluso, M., Cigna, D., Colucci, A. T., Modica, M. A., et al. (1993). γ-Interferon, Interleukin-4 and Interleukin-6 In Vitro Production in Old Subjects. *Autoimmunity* 16, 275–280. doi: 10.3109/08916939309014646.

Canela, A., Vera, E., Klatt, P., and Blasco, M. A. (2007). High-throughput telomere length quantification by FISH and its application to human population studies. *Proc. Natl. Acad. Sci.* 104, 5300–5305. doi: 10.1073/pnas.0609367104.

Deng, Y., Jing, Y., Campbell, A. E., and Gravenstein, S. (2004). Age-related impaired type 1 T cell responses to influenza: reduced activation ex vivo, decreased expansion in CTL culture in vitro, and blunted response to influenza vaccination in vivo in the elderly. *J. Immunol. Baltim. Md 1950* 172, 3437–3446. doi: 10.4049/jimmunol.172.6.3437.

Donato, A. J., Magerko, K. A., Lawson, B. R., Durrant, J. R., Lesniewski, L. A., and Seals, D. R. (2011). SIRT-1 and vascular endothelial dysfunction with ageing in mice and humans. *J. Physiol.* 589, 4545–4554. doi: 10.1113/jphysiol.2011.211219.

Gounder, S. S., Abdullah, B. J. J., Radzuanb, N. E. I. B. M., Zain, F. D. B. M., Sait, N. B. M., Chua, C., et al. (2018). Effect of Aging on NK Cell Population and Their Proliferation at Ex Vivo Culture Condition. *Anal. Cell. Pathol.* 2018, e7871814. doi: 10.1155/2018/7871814.

Herndon, F. J., Hsu, H.-C., and Mountz, J. D. (1997). Increased apoptosis of CD45RO− T cells with aging. *Mech. Ageing Dev.* 94, 123–134. doi: 10.1016/S0047-6374(97)01882-4.

Hwang, J. S., Hwang, J. S., Chang, I., and Kim, S. (2007). Age-Associated Decrease in Proteasome Content and Activities in Human Dermal Fibroblasts: Restoration of Normal Level of Proteasome Subunits Reduces Aging Markers in Fibroblasts From Elderly Persons. *J. Gerontol. Ser. A* 62, 490–499. doi: 10.1093/gerona/62.5.490.

Jelaska, A., and Korn, J. H. (1998). Anti-fas induces apoptosis and proliferation in human dermal fibroblasts: Differences between foreskin and adult fibroblasts. *J. Cell. Physiol.* 175, 19–29. doi: 10.1002/(SICI)1097-4652(199804)175:1<19::AID-JCP3>3.0.CO;2-F.

Li, X., Zhang, B., Wang, H., Zhao, X., Zhang, Z., Ding, G., et al. (2020). Aging affects responsiveness of peripheral blood mononuclear cells to immunosuppression of periodontal ligament stem cells. *J. Int. Med. Res.* 48, 0300060520930853. doi: 10.1177/0300060520930853.

Martínez-Zamudio, R. I., Dewald, H. K., Vasilopoulos, T., Gittens-Williams, L., Fitzgerald-Bocarsly, P., and Herbig, U. (2021). Senescence-associated β-galactosidase reveals the abundance of senescent CD8+ T cells in aging humans. *Aging Cell* 20, e13344. doi: 10.1111/acel.13344.

Miller, J. D., Ganat, Y. M., Kishinevsky, S., Bowman, R. L., Liu, B., Tu, E. Y., et al. (2013). Human iPSC-based Modeling of Late-Onset Disease via Progerin-induced Aging. *Cell Stem Cell* 13, 691–705. doi: 10.1016/j.stem.2013.11.006.

Papanagnou, E.-D., Terpos, E., Kastritis, E., Papassideri, I. S., Tsitsilonis, O. E., Dimopoulos, M. A., et al. (2018). Molecular responses to therapeutic proteasome inhibitors in multiple myeloma patients are donor-, cell type- and drug-dependent. *Oncotarget* 9, 17797–17809. doi: 10.18632/oncotarget.24882.

Phelouzat, M.-A., Laforge, T., Arbogast, A., Quadri, R. A., Boutet, S., and J. Proust, J. (1997). Susceptibility to apoptosis of T lymphocytes from elderly humans is associated with increased in vivo expression of functional Fas receptors. *Mech. Ageing Dev.* 96, 35–46. doi: 10.1016/S0047-6374(97)01883-6.

Phillip, J. M., Wu, P.-H., Gilkes, D. M., Williams, W., McGovern, S., Daya, J., et al. (2017). Biophysical and biomolecular determination of cellular age in humans. *Nat. Biomed. Eng.* 1, 1–12. doi: 10.1038/s41551-017-0093.

Potestio, M., Caruso, C., Gervasi, F., Scialabba, G., D'Anna, C., Di Lorenzo, G., et al. (1998). Apoptosis and ageing. *Mech. Ageing Dev.* 102, 221–237. doi: 10.1016/S0047-6374(98)00020-7.

Quinn, K. M., Fox, A., Harland, K. L., Russ, B. E., Li, J., Nguyen, T. H. O., et al. (2018). Age-Related Decline in Primary CD8+ T Cell Responses Is Associated with the Development of Senescence in Virtual Memory CD8+ T Cells. *Cell Rep.* 23, 3512–3524. doi: 10.1016/j.celrep.2018.05.057.

Rorteau, J., Chevalier, F. P., Bonnet, S., Barthélemy, T., Lopez-Gaydon, A., Martin, L. S., et al. (2022). Maintenance of Chronological Aging Features in Culture of Normal Human Dermal Fibroblasts from Old Donors. *Cells* 11, 858. doi: 10.3390/cells11050858.

Rübe, C. E., Bäumert, C., Schuler, N., Isermann, A., Schmal, Z., Glanemann, M., et al. (2021). Human skin aging is associated with increased expression of the histone variant H2A.J in the epidermis. *Npj Aging Mech. Dis.* 7, 1–11. doi: 10.1038/s41514-021-00060-z.

Sarkar, T. J., Quarta, M., Mukherjee, S., Colville, A., Paine, P., Doan, L., et al. (2020). Transient non-integrative expression of nuclear reprogramming factors promotes multifaceted amelioration of aging in human cells. *Nat. Commun.* 11, 1545–1545. doi: 10.1038/s41467-020-15174-3.

Schindowski, K., Leutner, S., Müller, W. E., and Eckert, A. (2000). Age-related changes of apoptotic cell death in human lymphocytes. *Neurobiol. Aging* 21, 661–670. doi: 10.1016/S0197-4580(00)00171-8.

Serra, J. A., Fernandez-Gutiérrez, B., Hernández-García, C., Vidan, M., Bañares, A., Ribera, J. M., et al. (1996). Early T-cell activation in elderly humans. *Age Ageing* 25, 470–478. doi: 10.1093/ageing/25.6.470.

Shinohara, S., Sawada, T., Nishioka, Y., Tohma, S., Kisaki, T., Inoue, T., et al. (1995). Differential Expression of Fas Antigen and Bcl-2 Protein on CD4+ T Cells, CD8+ T Cells, and Monocytes. *Cell. Immunol.* 163, 303–308. doi: 10.1006/cimm.1995.1130.

Slusher, A. L., Zúñiga, T. M., and Acevedo, E. O. (2019). Inflamm-Aging Is Associated with Lower Plasma PTX3 Concentrations and an Impaired Capacity of PBMCs to Express hTERT following LPS Stimulation. *Mediators Inflamm.* 2019, 2324193. doi: 10.1155/2019/2324193.

Wolf, J., Weinberger, B., Arnold, C. R., Maier, A. B., Westendorp, R. G. J., and Grubeck-Loebenstein, B. (2012). The effect of chronological age on the inflammatory response of human fibroblasts. *Exp. Gerontol.* 47, 749–753. doi: 10.1016/j.exger.2012.07.001.

Zheng, Y., Liu, X., Le, W., Xie, L., Li, H., Wen, W., et al. (2020). A human circulating immune cell landscape in aging and COVID-19. *Protein Cell* 11, 740. doi: 10.1007/s13238-020-00762-2.
